# MiR-30 promotes fatty acid beta-oxidation and endothelial cell dysfunction and is a circulating biomarker of coronary microvascular dysfunction in pre-clinical models of diabetes

**DOI:** 10.1186/s12933-022-01458-z

**Published:** 2022-02-24

**Authors:** Shawn Veitch, Makon-Sébastien Njock, Mark Chandy, M. Ahsan Siraj, Lijun Chi, HaoQi Mak, Kai Yu, Kumaragurubaran Rathnakumar, Carmina Anjelica Perez-Romero, Zhiqi Chen, Faisal J. Alibhai, Dakota Gustafson, Sneha Raju, Ruilin Wu, Dorrin Zarrin Khat, Yaxu Wang, Amalia Caballero, Patrick Meagher, Edward Lau, Lejla Pepic, Henry S. Cheng, Natalie J. Galant, Kathryn L. Howe, Ren-Ke Li, Kim A. Connelly, Mansoor Husain, Paul Delgado-Olguin, Jason E. Fish

**Affiliations:** 1 Department of Laboratory Medicine & Pathobiology, University of Toronto, Toronto, ON Canada; 2 Toronto General Hospital Research Institute, University Health Network, Toronto, ON Canada; 3 Stanford Cardiovascular Institute, Stanford University School of Medicine, Stanford, CA USA; 4 Translational Medicine, The Hospital for Sick Children, Toronto, ON Canada; 5 Keenan Biomedical Research Centre, Li Ka Shing Knowledge Institute, St. Michael’s Hospital, University of Toronto, Toronto, ON Canada; 6 Department of Medicine, Division of Cardiology, University of Colorado School of Medicine, Aurora, CO USA; 7 Princess Margaret Cancer Centre, University Health Network, Toronto, ON Canada; 8 Peter Munk Cardiac Centre, University Health Network, Toronto, ON Canada; 9 Department of Molecular Genetics, University of Toronto, Toronto, ON Canada

**Keywords:** Endothelial cell, Microvasculature, Diabetes, Extracellular vesicle, microRNA, Biomarker, Diastolic dysfunction, Heart failure with preserved ejection fraction

## Abstract

**Background:**

Type 2 diabetes (T2D) is associated with coronary microvascular dysfunction, which is thought to contribute to compromised diastolic function, ultimately culminating in heart failure with preserved ejection fraction (HFpEF). The molecular mechanisms remain incompletely understood, and no early diagnostics are available. We sought to gain insight into biomarkers and potential mechanisms of microvascular dysfunction in obese mouse (*db/db*) and lean rat (Goto-Kakizaki) pre-clinical models of T2D-associated diastolic dysfunction.

**Methods:**

The microRNA (miRNA) content of circulating extracellular vesicles (EVs) was assessed in T2D models to identify biomarkers of coronary microvascular dysfunction/rarefaction. The potential source of circulating EV-encapsulated miRNAs was determined, and the mechanisms of induction and the function of candidate miRNAs were assessed in endothelial cells (ECs).

**Results:**

We found an increase in miR-30d-5p and miR-30e-5p in circulating EVs that coincided with indices of coronary microvascular EC dysfunction (i.e., markers of oxidative stress, DNA damage/senescence) and rarefaction, and preceded echocardiographic evidence of diastolic dysfunction. These miRNAs may serve as biomarkers of coronary microvascular dysfunction as they are upregulated in ECs of the left ventricle of the heart, but not other organs, in *db/db* mice. Furthermore, the miR-30 family is secreted in EVs from senescent ECs in culture, and ECs with senescent-like characteristics are present in the *db/db* heart. Assessment of miR-30 target pathways revealed a network of genes involved in fatty acid biosynthesis and metabolism. Over-expression of miR-30e in cultured ECs increased fatty acid β-oxidation and the production of reactive oxygen species and lipid peroxidation, while inhibiting the miR-30 family decreased fatty acid β-oxidation. Additionally, miR-30e over-expression synergized with fatty acid exposure to down-regulate the expression of eNOS, a key regulator of microvascular and cardiomyocyte function. Finally, knock-down of the miR-30 family in *db/db* mice decreased markers of oxidative stress and DNA damage/senescence in the microvascular endothelium.

**Conclusions:**

MiR-30d/e represent early biomarkers and potential therapeutic targets that are indicative of the development of diastolic dysfunction and may reflect altered EC fatty acid metabolism and microvascular dysfunction in the diabetic heart.

**Supplementary Information:**

The online version contains supplementary material available at 10.1186/s12933-022-01458-z.

## Background

Type 2 diabetes (T2D) is characterized by system-wide metabolic changes, including insulin resistance and hyperglycemia [1]. Individuals with T2D have an elevated risk of developing heart failure (HF) [2, 3]. Indeed, > 40% of patients hospitalized with HF have T2D [4]. Heart failure includes both HF with reduced ejection fraction (HFrEF) and HF with preserved ejection fraction (HFpEF), with the latter being the predominant condition observed in T2D patients [5]. HFpEF is characterized by the stiffening of the left ventricle and impaired relaxation of the heart during diastole [6]. Notably, echocardiographic imaging has revealed that ~ 50% of diabetics without cardiac symptoms have measurable diastolic dysfunction, suggesting that there exists significant undiagnosed pathology in the diabetic population [7]. With a paucity of effective biomarkers for early detection, timely treatment of diastolic dysfunction and HFpEF remains a major challenge [8]. Notably, the EMPEROR-Preserved study recently reported that the SGLT2 inhibitor, empagliflozin, can reduce HF hospitalization and cardiovascular death in HFpEF patients with and without diabetes by ~ 21% [9]. This is the first drug that has been shown to be effective in the T2D HFpEF population. While this provides some optimism, we still lack a mechanistic understanding of HFpEF initiation and progression, hindering further progress.

Dysfunction of the coronary microvasculature has been implicated as a culprit in HFpEF. For example, HFpEF patients have defective coronary flow reserve (CFR) and increased microvascular resistance [10], and a reduction in the density of the microvasculature in the left ventricle [11]. Furthermore, microvascular dysfunction detected via compromised CFR is associated with the development of diastolic dysfunction and future risk of HFpEF [12]. While the mechanisms of microvascular dysfunction in HFpEF are poorly understood, T2D is associated with systemic inflammation, oxidative stress, cellular senescence and metabolic dysfunction, which can promote endothelial cell (EC) activation and vascular dysfunction [13].

Endothelial fatty acid (FA) metabolism may contribute to microvascular pathology, although the mechanisms have not been fully defined. In healthy conditions, ECs predominantly utilize glycolysis (~ 85%) for ATP production, with only minor contributions from FA β-oxidation (FAO) (~ 5%) and glucose oxidation [14]. In T2D, elevations in circulating levels of triglyceride-rich lipoproteins and saturated FAs [15], together with insulin resistance, enhances the utilization of FAs as a fuel source in cardiomyocytes, with detrimental consequences including lipotoxicity [16]. In the endothelium, excess FAs can promote FAO and this is associated with a myriad of detrimental effects, including insulin resistance, mitochondrial dysfunction, reactive oxygen species (ROS) production and oxidative stress, endoplasmic reticulum (ER) stress, inflammation, apoptosis, lipotoxicity, decreased eNOS activity and defective vasodilatory function [14, 17–20].

Extracellular vesicles (EVs) are increasingly recognized for their utility as circulating biomarkers of disease, including in diabetic cardiomyopathy [21]. EVs include exosomes (30–150 nm in diameter, produced by multivesicular bodies that are released by all cells), microvesicles (150–1000 nm, which bud off the plasma membrane preferentially as a result of cellular stress), and apoptotic bodies (1–5 µm, produced by apoptotic cells) [22, 23]. Secreted EVs contain molecular cargo (eg. protein, DNA, lipid, mRNA, lncRNA and miRNA) that can be taken up by neighbouring cells through binding, fusion, or endocytosis, altering recipient cellular function [21]. EVs can also be released into the circulation, where they can communicate with distant cells [24]. The abundance and type of EVs, along with their contents (including miRNA), vary during disease progression [25], thus providing a potential circulating biomarker to assess underlying health conditions. In the current study, we sought to identify early circulating biomarkers of HFpEF pathology by profiling circulating EV-encapsulated miRNAs. We found that miR-30 family members are upregulated in plasma EVs in rodent models of T2D, prior to the development of diastolic dysfunction, and that cardiac ECs with senescent-like characteristics may be the source of these EVs. Additionally, we determined that miR-30 enhances FAO in ECs, which promotes oxidative stress and suppresses eNOS expression. Finally, knock-down of miR-30 in *db/db* mice reduced markers of oxidative stress and DNA damage/senescence in the coronary microvasculature. Our findings therefore suggest that miR-30 is a putative biomarker and effector of microvascular dysfunction in HFpEF pathogenesis.

## Methods

A complete description of Methods is included in the Additional File 1: Data supplement.

### Experiments involving animals

All animal use protocols (AUPs) for mouse and rat experiments were approved by the Animal Care Committees at the University Health Network (Toronto) and St. Michael’s Hospital (Toronto), respectively, and adhere to the Canadian Council on Animal Care (CCAC) guidelines and the NIH Guide for the Care and Use of Laboratory Animals. For mouse experiments, inhaled 0.5–3% isoflurane vapour was used for procedures. For euthanasia, mice were given > 5% isoflurane with a vaporizer until respiration ceased and were then exsanguinated under anesthesia. For mice that were not needed for experiments, mice were given inhaled 0.5–3% isoflurane followed by CO_2_ asphyxiation (fill rate of 20–30% volume/minute) under anaesthesia. For rat experiments, isoflurane at 0.5–1% was inhaled during anaesthesia for echocardiography and pressure–volume loop analyses. All rats were euthanized following cardiac catheterization, while under anaesthesia (2% isoflurane) via cervical dislocation.

### Statistical analysis

All experiments were performed at least 3 independent times on biological replicates unless otherwise stated. The exact number of replicates is stated in the figure legends or indicated in the figures as individual data points. Data plots depict the mean ± standard error of the mean unless stated otherwise. Statistical analysis was performed in GraphPad Prism (version 9.2.0) using an unpaired two-sided Student’s t-test for pairwise comparisons, or One-Way ANOVA with Holm-Sidak’s multiple comparisons test for multiple comparisons, unless stated otherwise. For all figures, *, ** and *** indicate p < 0.05, p < 0.01 and p < 0.001, respectively.

## Results

### Cardiac microvascular rarefaction precedes the development of diastolic dysfunction in pre-clinical T2D models

**Fig. 1:**
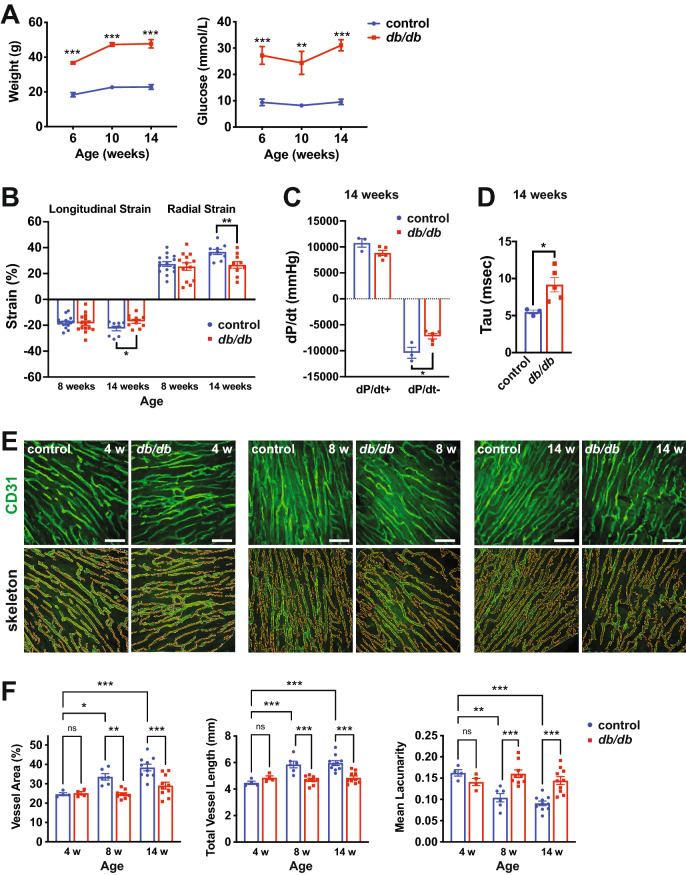
Diabetic mice develop diastolic dysfunction accompanied by microvascular rarefaction in the left ventricle. **A** Fasting blood glucose levels and body weight in *db/db* mice and *db/* + controls at 6, 10 and 14 weeks of age. n = 4–5 mice per group. ** and *** indicate p < 0.01 and p < 0.001, respectively, for *db/db* vs. *db/* + controls at the specified timepoint using an unpaired t-test. **B** Longitudinal and radial strain analysis at 8 and 14 weeks in *db/db* mice and *db/* + controls. * and ** indicate p < 0.05 and p < 0.01, respectively for *db/db* vs. *db/* + controls at the specified timepoint using an unpaired t-test. **C** Pressure–Volume (PV) loop analysis at 14 weeks in *db/db* mice and *db/* + controls, depicting the dP/dt maximum (dP/dt +) and minimum (dP/dt-) values. * indicates p < 0.05 for *db/db* vs. *db/* + using an unpaired t-test. **D** Tau measurements, indicative of the exponential decay of the ventricular pressure during isovolumetric relaxation, from PV loop analysis at 14 weeks in *db/db* mice and *db/* + controls. * indicates p < 0.05 for *db/db* vs. *db/* + using an unpaired t-test. **E** 2-photon confocal microscopy of cardiac microvasculature in the left ventricle as assessed by CD31 immunofluorescence at 4, 8 and 14 weeks in *db/db* mice and *db/* + controls. The top images are CD31 immunofluorescence and the bottom are the skeleton outlines of the microvasculature. Representative images are shown. Scale bar = 45 μm. **F** Quantification of microvascular density, as assessed by measurement of microvascular area, total vessel length, and mean lacunarity in 4-, 8- and 14-week *db/db* and *db/* + control mice. Each data point represents the mean of multiple fields of view from one mouse. *, ** and *** indicate p < 0.05, p < 0.01 and p < 0.001, respectively for the specified comparisons using ANOVA with Holm-Sidak multiple comparisons test. NS = not significant. All data in the figure depict mean ± SEM

T2D in humans and animal models is associated with EC dysfunction and microvascular rarefaction in the left ventricle of the heart [26]. This is thought to impact cardiac function, resulting in impaired relaxation during diastole [11]. We characterized cardiac function in two genetic models of T2D. Leptin-receptor mutant mice (*Lepr*^*db/db*^; also known as *db/db*) are obese and hyperglycemic (Fig. 1A), while Goto-Kakizaki (GK) rats are lean and hyperglycemic (Additional File 1: Fig. S1B). Echocardiography was performed in diabetic (*db/db*) mice and non-diabetic controls (*db*/ +) at 8 and 14 weeks of age with traditional and strain measurements. As measured by echocardiographic analysis of strain, significant diastolic dysfunction was present in *db/db* mice at 14 weeks of age, but not at 8 weeks of age, suggesting that defective diastolic relaxation occurs at later stages of disease in *db/db* mice (Fig. 1B). Pressure–volume (PV) loop analysis corroborated these findings at 14 weeks, as indicated by altered dP/dt- (Fig. 1C) and Tau logistic (Fig. 1D) values. Systolic function was normal at 14 weeks, as demonstrated by unchanged ejection fraction (EF), fractional shortening (FS), LV mass and LV diastolic volume (Additional File 1: Fig. S1A). Heart rate (HR) was also not significantly different at 14 weeks (Additional File 1: Fig. S1A). Cardiac function was similarly assessed in lean diabetic GK rats and wild-type Wistar (WS) controls at 28 weeks of age, a relatively advanced stage of T2D. Echocardiographic analysis of strain (Additional File 1: Fig. S1C) and PV loop analysis (Additional File 1: Fig. S1D, E) revealed the presence of diastolic dysfunction in GK rats at this time-point. No defects in systolic parameters were observed (Additional File 1: Fig. S1F). The *db/db* mouse model and the GK rat model therefore represent obese and lean T2D-associated diastolic dysfunction models, respectively.

We next assessed microvascular density in the left ventricle in *db/db* mice and non-diabetic *db/* + controls at 4, 8 and 14 weeks using 2-photon confocal microscopy of CD31-stained sections. We observed a significant decrease in total vessel area and total vessel length, as well as an increase in mean lacunarity, a measure of the spaces between microvessels, at 8 and 14 weeks, but not at 4 weeks (Fig. 1E, F). Importantly, microvascular rarefaction was first evident at 8 weeks in *db/db* mice, a time-point that preceded overt diastolic dysfunction. Coronary microvascular density was likewise reduced in 28-week GK rats compared to Wistar controls (Additional File 1: Fig. S2), a time-point that was coincident with diastolic dysfunction.

### Pre-clinical models of T2D-associated diastolic dysfunction have altered circulating extracellular vesicle characteristics

**Fig. 2:**
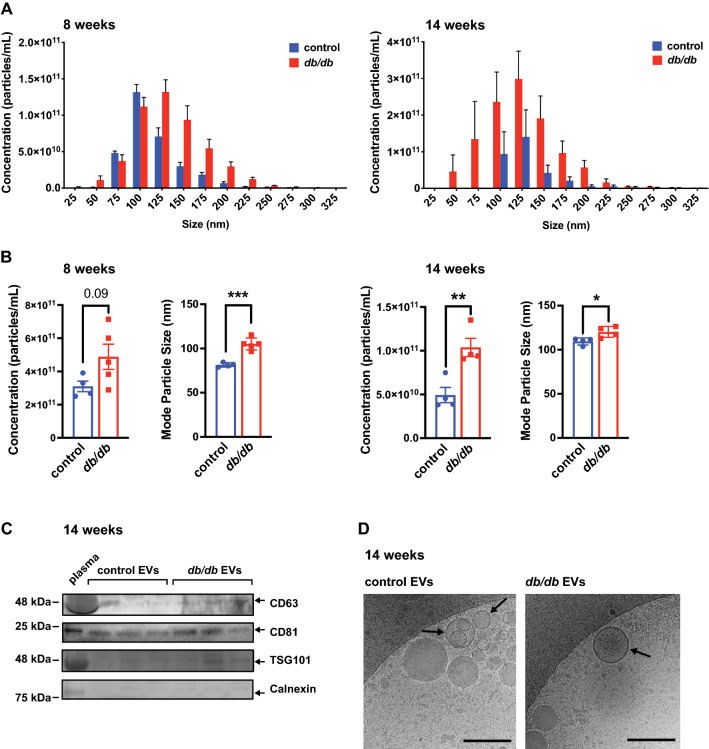
Circulating extracellular vesicles are increased in size and concentration in T2D mice. **A** Nanoparticle tracking analysis (NTA) of EV concentration binned by size from samples isolated from an equal volume of plasma from *db/db* and *db/* + controls at 8 and 14 weeks. n = 4–5 samples per group. **B** Quantification of EV concentration across all size bins and mode particle size in 8- and 14-week *db/db* and *db/* + control mice. *, ** and *** indicate p < 0.05, p < 0.01 and p < 0.001, respectively, for *db/db* vs. *db/* + at the specified timepoint using an unpaired t-test. **C** Western blotting of EV markers (CD63, CD81 and TSG101) and a non-EV marker (Calnexin) in EV samples and a total plasma control from 3 *db/db* and 3 *db/* + control mice at 14 weeks. The position of molecular weight markers is indicated to the left and arrows to the right indicate the correct band. **D** Cryo-transmission scanning electron microscopy of EV samples from *db/db* and *db/* + controls at 14 weeks. A representative image is shown. Arrows indicate examples of EV structures. Scale bar = 200 nm. All data in the figure depict mean ± SEM

To identify potential circulating biomarkers of diastolic dysfunction, we first determined whether the size and/or concentration of circulating EVs were altered during the progression of T2D in mouse and rat models. Circulating EVs were isolated from equal volumes of plasma using ExoQuick reagent. Nanoparticle tracking analysis (NTA) revealed a significant increase in EV size in 8 week *db/db* mice and an increase in both size and concentration at 14 weeks compared to their respective *db/* + controls (Fig. 2A, B). Similarly, there was an increase in size and concentration of EVs in the plasma of GK rats compared to Wistar controls at 28 weeks (Additional File 1: Fig. S3). The presence of EVs was confirmed in *db/db* and control *db/* + samples at 14 weeks via western blotting for the EV markers CD63, CD81 and TSG101 (Fig. 2C). The EV preparation had minimal staining for the ER marker Calnexin, as expected. EVs were also visualized by cryo-transmission scanning electron microscopy in *db/db* and control samples at 14 weeks (Fig. 2D). Taken together, EV characteristics are altered in the circulation of T2D models, prior to, and coincident with, diastolic dysfunction.

### miRNAs are differentially abundant in circulating EVs in pre-clinical models of T2D-associated diastolic dysfunction

**Fig. 3:**
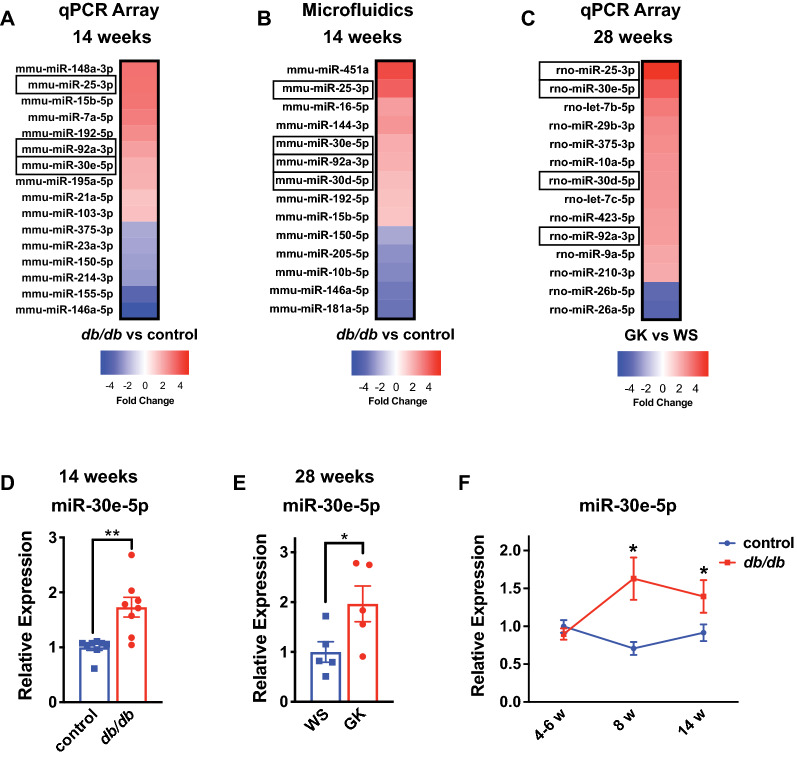
Extracellular vesicle miRNAs are differentially abundant in T2D models. **A** Heat map of differentially abundant miRNAs (unpaired t-test) in EVs isolated from *db/db* mice and *db/* + controls at 14 weeks using a qRT-PCR array. n = 4. **B** Heat map of differentially abundant miRNAs (unpaired t-test) in EVs isolated from *db/db* mice and *db/* + controls at 14 weeks using a microfluidics platform. n = 4. **C** Heat map of differentially abundant miRNAs in EVs isolated from Goto-Kakizaki (GK) rats and Wistar (WS) controls at 28 weeks using a qRT-PCR array. n = 3. In panels A-C, miR-25-3p, miR-30d-5p, miR-30e-5p, miR-92a-3p are highlighted, as these were common among platforms and between mouse and rat models of T2D. **D** qRT-PCR validation of miR-30e-5p expression in EVs isolated from *db/db* mice and *db/* + controls at 14 weeks. Data is relative to the mean of the control group. ** indicates p < 0.01 for *db/db* vs. *db/* + using an unpaired t-test. **E** qRT-PCR validation of miR-30e-5p expression in EVs isolated from GK rats and WS controls at 28 weeks. Data is relative to the mean of the WS control. * indicates p < 0.05 for GK vs. WS using an unpaired t-test. **F** Time-course analysis of miR-30e-5p expression in *db/db* mice and *db/* + controls at 4–6, 8 and 14 weeks. Data is relative to the mean of the control 4–6 week time-point group. n = 4–5 mice per time-point and genotype. * indicates p < 0.05 for *db/db* vs. *db/* + at the specified timepoint using an unpaired t-test. See Additional File 1: Fig. S4 for individual data points for the heat maps in (**A**), (**B**) and (**C**). All data in the figure depict mean ± SEM

We next profiled the miRNA content of circulating plasma EVs. We used a SYBR Green quantitative reverse transcriptase PCR (qRT-PCR) miRNA array to measure 84 miRNAs—chosen based on their presence in serum, plasma, and other extracellular bodily fluids, and their differential regulation in disease, including diabetes and heart disease (Additional File 2: Table S1). We identified 16 dysregulated miRNAs (10 up-regulated and 6 down-regulated) in EVs from *db/db* mice at 14 weeks compared to *db/* + controls (Fig. 3A, Additional File 1: Fig. S4A). This time-point was chosen because of the presence of significant coronary microvascular rarefaction and diastolic dysfunction. To validate these findings, we utilized an independent methodology on a distinct set of EV samples from *db/db* mice and *db/* + controls at 14 weeks. A microfluidics platform that utilizes locked nucleic acid (LNA) primer sets to simultaneously measure 96 miRNAs (Additional File 2: Table S1) confirmed that 7 of the 16 miRNAs identified using the SYBR Green array platform, including miR-25-3p, miR-30e-5p and miR-92a-3p were differentially abundant (Fig. 3B, Additional File 1: Fig. S4B). In addition, the microfluidics platform revealed that miR-30d-5p was differentially regulated, but other miR-30 family members (miR-30a, b, c) were unchanged (Fig. 3B, Additional File 1: Fig. S4B and data not shown). To determine which of these miRNAs might be similarly dysregulated in an independent model of T2D-associated diastolic dysfunction, we assessed miRNAs in EVs isolated from the plasma of GK rats at 28 weeks using qRT-PCR miRNA arrays (Fig. 3C, Additional File 1: Fig. S4C). Importantly, several miRNAs were similarly increased in circulating EVs in both mouse and rat models of T2D-associated diastolic dysfunction, including miR-25-3p, miR-30d-5p, miR-30e-5p and miR-92a-3p (Fig. 3A–C). Validation of the expression of miR-30e-5p using qRT-PCR confirmed the upregulation of this miRNA in distinct plasma EV samples from both mouse and rat models of T2D (Fig. 3D, E).

To determine whether the identified miRNAs might be early markers of HFpEF pathogenesis, we measured their levels across multiple time points in *db/db* mice and their respective *db/* + controls. We found that miR-30e-5p (Fig. 3F) and several other miRNAs (Additional File 1: Fig. S5) were induced as early as 8 weeks of age, preceding the development of diastolic dysfunction, but coincident with coronary microvascular rarefaction. In contrast, miR-25-3p was induced only at later stages (i.e. 14 weeks). We did not detect changes in miR-92a-3p in these samples (Additional File 1: Fig. S5). Taken together, we identified several miRNAs that were elevated in EVs at early stages of HFpEF pathogenesis, and in particular, miR-30d-5p and miR-30e-5p were induced in both mouse and rat models of T2D-associated microvascular rarefaction and diastolic dysfunction.

### miR-30d and miR-30e are upregulated in cardiac endothelium in T2D mice

**Fig. 4:**
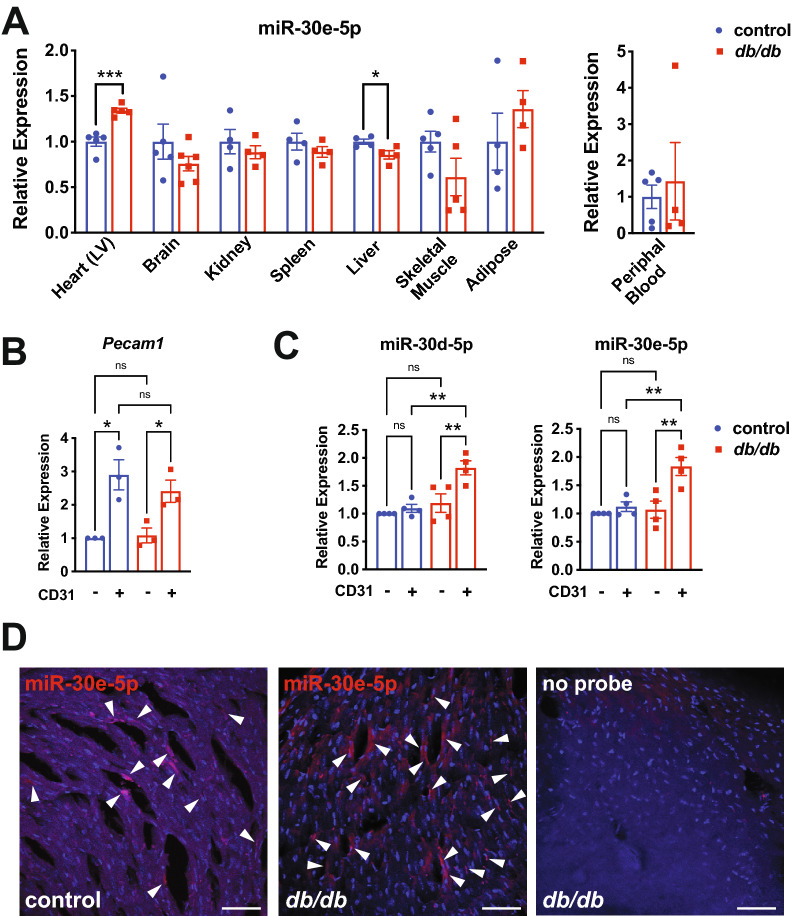
miR-30d-5p and miR-30e-5p are upregulated in the cardiac endothelium in T2D mice. **A** qRT-PCR of miR-30e-5p in the specified tissues and peripheral blood mononuclear cells in *db/db* mice and *db/* + controls at 14 weeks of age. Data is relative to the mean of the control group for each tissue sample. * and *** indicate p < 0.05 and p < 0.001, respectively, for *db/db* vs. *db/* + in the specified tissue using an unpaired t-test. **B** Confirmation of EC enrichment of CD31^+^ cells isolated from the heart in *db/db* and *db/* + controls at 14 weeks of age by qRT-PCR of *Pecam1* mRNA. Data is relative to the mean of the CD31^−^ control group. * indicates p < 0.05 for the specified comparisons using ANOVA with Holm-Sidak multiple comparisons test. NS = not significant. **C** miR-30d-5p and miR-30e-5p are specifically upregulated in the CD31^+^ endothelium of the heart of *db/db* mice at 14 weeks of age compared to *db/* + controls. Data is relative to the mean of the CD31^−^ control group. ** indicates p < 0.01 for the specified comparisons using ANOVA with Holm-Sidak multiple comparisons test. NS = not significant. **D** Confocal imaging of miRNAscope with a miR-30e-5p probe in left ventricles of *db/db* and control *db/* + mice at 14 weeks. A ‘no probe’ control is shown to the right. Arrows indicate regions of expression that are presumed to be endothelial based on cell morphology and anatomy. Representative images are shown. Scale bar = 50 μm. All data in the figure depict mean ± SEM

Circulating EVs can originate from circulating cells and various tissue sources. To define the potential origin of circulating EV miRNAs that were elevated in T2D mice, we measured miR-25-3p, miR-30e-5p and miR-92a-3p in a variety of tissues, including heart, brain, kidney, spleen, liver, skeletal muscle and visceral white adipose tissue at 14 weeks of age (Fig. 4A and Additional File 1: Fig. S6). We also measured miR-30e-5p in circulating peripheral blood cells (Fig. 4A). Interestingly, miR-30e-5p was significantly upregulated in the left ventricle of the heart and was down-regulated in liver but was not altered in other tissues (Fig. 4A). MiR-25-5p and miR-92a-3p were not differently expressed across tissues, although levels trended upwards in several tissues (Additional File 1: Fig. S6). We next isolated ECs from the left ventricle of the heart using fluorescence-activated cell sorting of CD31^+^ cells and confirmed the enrichment of *Pecam1* mRNA (an EC marker) in these samples (Fig. 4B). Importantly, we observed a significant upregulation of miR-30d-5p and miR-30e-5p in *db/db* CD31^+^ cardiac ECs, but not in CD31^−^ cells (Fig. 4C). Additionally, we used miRNAscope to visualize miR-30e-5p expression in the left ventricle in 14-week-old *db/db* and control *db/* + mice. Expression was enriched in the microvascular endothelium and endocardium of *db/db* mice, with lower levels of expression in non-endothelial cells (Fig. 4D). Staining for miR-30e-5p was lower in *db/* + control mice. Taken together, these results imply that cardiac ECs from diabetic mice may be a source of circulating miR-30d/e-containing EVs, suggesting that circulating levels of this miRNA may be indicative of microvascular dysfunction in the heart.

### Senescent ECs have elevated production and secretion of miR-30d and miR-30e

**Fig. 5:**
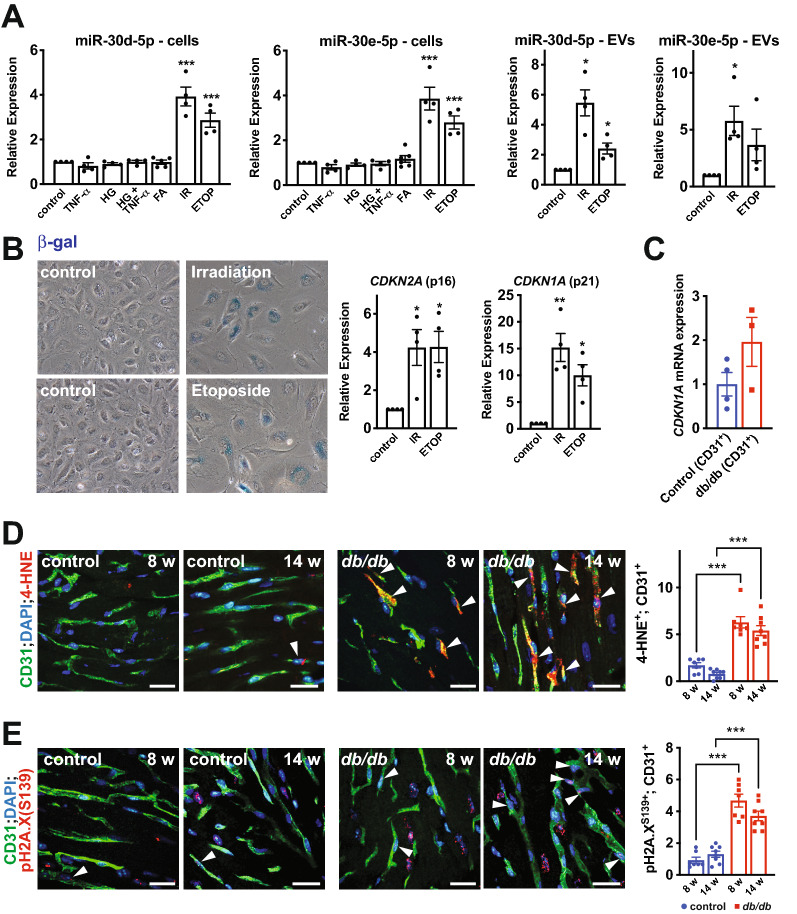
Senescence pathways induce miR-30d/e expression and secretion from ECs. **A** Left; qRT-PCR of miR-30d and miR-30e in cultured human umbilical vein ECs (HUVECs) exposed to diabetic stimuli (i.e. TNFα (10 ng/mL), high glucose [HG] (20 mM), TNFα + high glucose or the fatty acid, palmitate [FA] (40 μM)) or senescence-inducing stimuli (i.e. irradiation [IR] or etoposide [ETOP]). *** indicates p < 0.001 for the specified stimulus vs. vehicle control using ANOVA with Holm-Sidak multiple comparisons test. Right; secretion of miR-30d or miR-30e as assessed by qRT-PCR of EVs secreted by HUVECs exposed to senescence-inducing stimuli. * indicates p < 0.05 for the specified stimulus vs. vehicle control using ANOVA with Holm-Sidak multiple comparisons test. Data is relative to the control for each independent experiment. **B** Representative staining for senescence-associated β-gal activity and qRT-PCR for *CDKN2A* (p16) and *CDKN1A* (p21) expression in HUVECs exposed to irradiation [IR] or treated with etoposide [ETOP]. Data is relative to the control for each independent experiment. **C** qRT-PCR of *CDKN1A* (p21) expression in CD31^+^ cells isolated from *db/db* and *db/* + control hearts at 14 weeks of age. Representative images of immunofluorescent staining for 4-HNE and CD31 (**D**) and pH2A.X (S139) and CD31 (**E**) in the left ventricle of 8- and 14-week *db/db* mice and *db/* + controls. Scale bar = 22 μm. Arrowheads indicate examples of 4-HNE^+^ or pH2A.X^+^ ECs. Quantification of 4-HNE^+^;CD31^+^ double-positive ECs and pH2A.X^+^;CD31^+^ double-positive ECs per field of view is shown to the right. Each data point represents the mean of multiple fields of view from one mouse. *** indicates p < 0.001 for the specified comparison using ANOVA with Holm-Sidak multiple comparisons test. All data in the figure depict mean ± SEM

We next sought to assess the potential mechanisms of miR-30d/e up-regulation in the endothelium of diabetic mice. To this end, we exposed cultured ECs to conditions that mimic the diabetic microenvironment (Fig. 5A). This included 24 h treatment with pro-inflammatory cytokines (10 ng/ml TNFα), high glucose (25 mM of D-glucose), a combination of TNFα and 25 mM D-Glucose, or long chain free FAs (40 μM of palmitate). However, none of these stimuli induced miR-30d/e expression (Fig. 5A). Since senescence has been implicated in diabetic microvascular pathology [27–30], and previous studies in cancer cells have shown that DNA damage and senescence pathways can induce miR-30e expression (31, 32), we induced senescence in cultured ECs by exposure to ionizing radiation (IR) or treatment with etoposide (ETOP), which drive oxidative stress- and DNA damage-dependent senescence [33, 34]. Senescence was confirmed by staining for senescence-associated β-gal activity and expression of *CDKN1A* (p21) and *CDKN2A* (p16) (Fig. 5B) [35]. Senescence-inducing stimuli robustly increased the expression of miR-30d and miR-30e in ECs and promoted their secretion in EVs (Fig. 5A).

We next sought to further establish if the ECs in the diabetic heart have senescent-like characteristics. To this end, we measured *CDKN1A* (p21) mRNA levels in CD31^+^ cells isolated from *db/db* and control *db/* + hearts at 14 weeks of age and found a twofold increase in expression in diabetic ECs (Fig. 5C). We also assessed levels of 4-hydroxy-2-nonenal (4-HNE), a lipid peroxidation product that is produced from n-6 polyunsaturated FAs in highly oxidative environments and which has been implicated in driving cellular senescence [34]. Levels of 4-HNE were highly upregulated in microvascular ECs in the left ventricle of *db/db* mice at both 8 and 14 weeks of age (Fig. 5D). Furthermore, we assessed levels of phospho-H2A.X (Ser139), also known as γH2A.X, a marker of DNA damage, telomere shortening and cellular senescence [36, 37]. Levels of γH2A.X were markedly elevated at 8 and 14 weeks of age in the endothelium of *db/db* mice (Fig. 5E). Taken together, these results are suggestive of microvascular dysfunction and senescent-like characteristics in the endothelium of the left ventricle in diabetic mice, as early as 8 weeks of age, prior to the development of diastolic dysfunction. Thus, oxidative stress, DNA damage and senescence pathways may be involved in the induction of miR-30d/e expression and secretion from diabetic ECs.

### miR-30d/e regulate fatty acid biosynthesis and metabolism genes in ECs

**Fig. 6:**
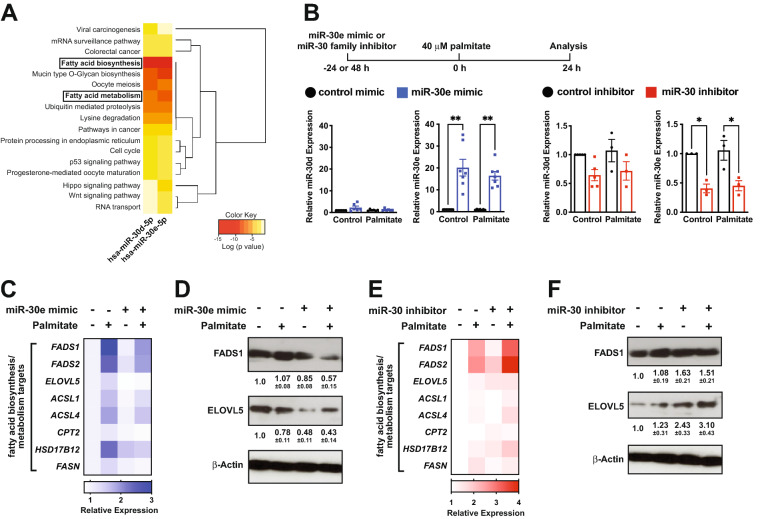
miR-30 regulates a network of genes that are involved in fatty acid biosynthesis and metabolism pathways. **A** KEGG Pathway analysis (DIANA-miRPath v3.0 with Tarbase v7.0) of human miR-30d-5p and miR-30e-5p target genes reveals a significant enrichment in fatty acid biosynthesis and fatty acid metabolism genes. Pathway analysis in mouse is shown in Additional File 1: Fig. S7. **B** Top; schematic of experimental approach to assess the function of miR-30 in cultured ECs. Bottom; qRT-PCR of miR-30d and miR-30e in cells transfected with miR-30e mimic (left) or a miR-30 family LNA inhibitor (right) compared to their respective controls. Data is relative to the unstimulated control for each independent experiment. * and ** indicate p < 0.05 and p < 0.01, respectively, for the specified comparison using ANOVA with Holm-Sidak multiple comparisons test. **C** Heat map of changes in gene expression of putative miR-30 target genes in fatty acid biosynthesis/metabolism pathways by qRT-PCR in ECs over-expressing miR-30e under control or palmitate-stimulated conditions. The mean of multiple independent experiments is indicated. **D** Representative western blots of FADS1, ELOVL5 and β-actin loading control in ECs over-expressing miR-30e with or without palmitate treatment. Densitometry is included below from n = 3 independent experiments (mean ± SEM). **E** Heat map of changes in gene expression of putative miR-30 target genes by qRT-PCR in ECs transfected with miR-30 family LNA inhibitor under control or palmitate-stimulated conditions. The mean of multiple independent experiments is indicated. **F** Representative western blots of FADS1, ELOVL5 and β-actin loading control in ECs transfected with miR-30 family inhibitor. Densitometry is included below from n = 3 independent experiments (mean ± SEM). See Additional File 1: Fig. S8 for individual data points and statistical analysis of data presented as heatmaps in (**C**) and (**E**). All data in the figure depict mean ± SEM

To uncover the potential function of miR-30d-5p and miR-30e-5p in the endothelium, we performed KEGG pathway analysis of target genes using prediction software (DIANA-miRPath v3.0 with Tarbase v7.0) [38]. This revealed a significant over-representation of genes involved in fatty acid biosynthesis and metabolism pathways in humans (Fig. 6A). Predicted miR-30d/e binding sites were identified in a number of genes involved in fatty acid biosynthesis and metabolism pathways, including *FADS1*, *FADS2*, *ELOVL5*, *ACSL1*, *ACSL4*, *CPT2*, *HSD17B12* and *FASN*. Fatty acid biosynthesis and fatty acid metabolism were also significantly enriched in mice, with *Ascl6, Elovl5* and *Fasn* being predicted targets (Additional File 1: Fig. S7). We used miRTarBase v8.0 [39] to examine whether these targets have previously been validated and found experimental evidence that *ELOVL5*, *ACSL4* and *HSD17B12* are targeted by the miR-30 family in human cells. As miR-30d and miR-30e differ by only two nucleotides, which are outside of the seed sequence, they are predicted to target nearly identical sets of genes.

To confirm whether the predicted target genes are indeed regulated, we overexpressed miR-30e using a miRNA mimic or knocked-down the miR-30 family using a miR-30 family locked nucleic acid (LNA) inhibitor in cultured human umbilical vein ECs (HUVEC) and then exposed the cells to 40 µM palmitate to activate fatty acid metabolic pathways. qRT-PCR and western blotting were used to assess expression of miR-30 target genes (Fig. 6B–F). We first confirmed the efficiency and specificity of miR-30 over-expression and knock-down (Fig. 6B). Compared to control mimic transfection, ECs transfected with the miR-30e mimic had robust increases in miR-30e, but not miR-30d, confirming the specificity of the qRT-PCR reagents (Fig. 6B). Transfection with a miR-30 family LNA inhibitor significantly decreased miR-30e levels by ~ 55–60%, but there was a more modest ~ 30% reduction in miR-30d, which did not reach statistical significance (Fig. 6B). In unstimulated cells, over-expression of miR-30e repressed the expression of *ELOVL5*, mRNA but not other predicted target genes (Fig. 6C; Additional File 1: Fig. S8A). However, palmitate stimulation led to the induction of the mRNAs encoding the majority of the fatty acid biosynthesis/metabolism genes that were identified as potential miR-30 targets, and their expression was blunted in the presence of miR-30e mimic (Fig. 6C; Additional File 1: Fig. S8A). Western blotting confirmed the miR-30e-dependent repression of two of the predicted targets, FADS1 and ELOVL5, at the protein level (Fig. 6D). Meanwhile, despite the partial knock-down of the miR-30 family, several predicted targets (e.g. *FADS2, ELOVL5, FASN*) were increased at the mRNA level in unstimulated cells, and additional target genes were significantly elevated or showed a trend towards increased expression in palmitate-stimulated cells transfected with miR-30 inhibitor (Fig. 6E; Additional File 1: Fig. S8B). The up-regulation of FADS1 and ELOVL5 in miR-30 knock-down cells was confirmed at the protein level by western blotting (Fig. 6F). Taken together, our results reveal that miR-30 regulates a network of fatty acid biosynthesis/metabolism genes in ECs.

### miR-30e enhances fatty acid β-acid oxidation, promoting oxidative stress and EC dysfunction in vitro

Since miR-30d/e regulate fatty acid biosynthesis and metabolism pathway components, we investigated the impact of modulating miR-30 levels on EC responses to exposure to free fatty acids (FFAs). Notably, circulating levels of FFAs, including palmitate, are greatly elevated in T2D, and can drive EC dysfunction [15, 19]. Recently, lipid droplets were shown to dynamically form and disassemble in ECs following exposure to FFAs [40]. We transfected HUVEC with control or miR-30e mimic and assessed lipid droplet formation by BODIPY staining after a 24 h exposure to 40 μM palmitate or 40 μM oleate (Additional File 1: Fig. S9A). In cells transfected with control mimic, oleate stimulated lipid droplet formation, but palmitate did not, consistent with previous studies [40]. Interestingly, miR-30e mimic transfection increased lipid droplet formation even in vehicle-treated cells, and this was further enhanced in palmitate and oleate-treated cells. This suggests that miR-30e may enhance storage of FAs in lipid droplets. Lipid droplets protect the endothelium from lipotoxicity [40], which can induce ER stress [41]. We measured the spliced isoform of XBP1 as an index of endoplasmic reticulum (ER) stress [42]. While palmitate treatment increased sXBP1 levels, modulation of miR-30 did not significantly impact these levels (Additional File 1: Fig. S9B).

**Fig. 7:**
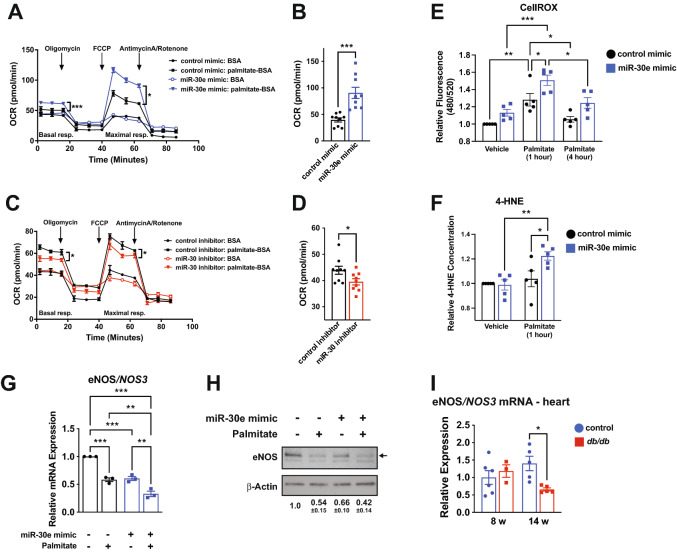
miR-30 enhances exogenous fatty acid β-oxidation and promotes oxidative stress and EC dysfunction. **A** Representative Seahorse tracing of oxygen consumption rate (OCR) in control and miR-30e over-expressing ECs. Basal and maximal respiration of exogenous, but not endogenous FA, was enhanced in miR-30e over-expressing cells. Error bars depict 2–3 technical replicates from a representative experiment. * and *** indicate p < 0.05 and p < 0.001, respectively, for the specified comparison using ANOVA with Holm-Sidak multiple comparisons test. Technical replicates were averaged and the value at each timepoint for basal and maximal respiration was used for paired statistical analyses (n = 3). **B** Quantification of exogenous FA oxidation (maximum OCR after FCCP injection minus minimum OCR after AntA/rotenone) in ECs over-expressing miR-30e. n = 3 independent experiments with 3 technical replicates. *** indicates p < 0.001 for miR-30e mimic vs. control mimic using an unpaired t-test. **C** Representative Seahorse tracing of OCR in control and miR-30 knock-down ECs. Basal and maximal respiration of exogenous FA was reduced by miR-30 knock-down. Error bars depict 2–3 technical replicates from a representative experiment. * indicates p < 0.05 for the specified comparisons using ANOVA with Holm-Sidak multiple comparisons test. Technical replicates were averaged and the value at each timepoint for basal and maximal respiration was used for paired statistical analyses (n = 3). **D** Quantification of exogenous FA oxidation in ECs with miR-30 knock-down. n = 3 independent experiments with 3 technical replicates. * indicates p < 0.05 for miR-30 inhibitor vs. control inhibitor using an unpaired t-test. **E** Measurement of reactive oxygen species (ROS) in ECs transfected with control or miR-30e mimic exposed to palmitate. Basal and palmitate-induced ROS production was increased in miR-30e over-expressing cells. Data is relative to the unstimulated control for each independent experiment. *, ** and *** indicate p < 0.05, p < 0.01 and p < 0.001, respectively, for the specified comparisons using ANOVA with Holm-Sidak multiple comparisons test. NS = not significant. **F** 4-HNE measurements in vehicle or palmitate stimulated ECs transfected with control or miR-30e mimic. Data is relative to the unstimulated control for each independent experiment. * and ** indicate p < 0.05 and p < 0.01, respectively for the specified comparisons using ANOVA with Holm-Sidak multiple comparisons test. **G** qRT-PCR measurement of eNOS/*NOS3* mRNA in cultured ECs transfected with control or miR-30e mimic in the presence or absence of palmitate. Data is relative to the unstimulated control for each independent experiment. ** and *** indicate p < 0.01 and p < 0.001, respectively, for the specified comparisons using ANOVA with Holm-Sidak multiple comparisons test. **H** Representative western blot demonstrating a reduction in eNOS protein in ECs over-expressing miR-30e and exposed to palmitate. Densitometry is included below from n = 5 independent experiments (mean ± SEM). **I** qRT-PCR measurement of eNOS/*NOS3* mRNA in the left ventricle of *db/db* mice and *db/* + controls at 8 and 14 weeks of age. Data is relative to the mean of control samples at 8 weeks of age. * indicates p < 0.05 for the specified comparison using ANOVA with Holm-Sidak multiple comparisons test. All data in the figure depict mean ± SEM

We next tested whether miR-30 regulates endogenous or exogenous FA β-oxidation (FAO) through measurement of the cellular oxygen consumption rate (OCR) with Seahorse XF Analyzer. MiR-30e overexpression significantly increased basal and maximal mitochondrial oxidation of exogenous palmitate, but did not affect endogenous FAO (Fig. 7A, B), while knockdown of the miR-30 family significantly decreased basal and maximal oxidation of exogenous palmitate (Fig. 7C, D). This data suggests that miR-30 promotes FAO in ECs. Utilization of FAs as a fuel source has previously been implicated in the generation of ROS and oxidative stress [17, 43]. Measurement of ROS using CellROX revealed that miR-30e over-expression enhanced ROS production in cells exposed to palmitate (Fig. 7E). The presence of oxidative stress was confirmed by measurement of 4-HNE, a lipid peroxidation product that is formed in highly oxidative environments, which revealed enhanced levels in miR-30e over-expressing cells exposed to palmitate (Fig. 7F). ROS production and oxidative stress is associated with impairment of endothelial nitric oxide synthase (eNOS) expression and activity [44, 45]. Importantly, miR-30e overexpression reduced eNOS/*NOS3* mRNA expression basally, and exposure to palmitate resulted in an even greater reduction, both at the level of mRNA (Fig. 7G) and protein (Fig. 7H). Finally, we measured eNOS/*NOS3* mRNA levels in the left ventricle from 8- and 14-week *db/db* and control *db/* + mice. Despite the vascular rarefaction that occurs at 8 weeks of age, levels of eNOS/*NOS3* mRNA were unchanged, suggesting that there may be a compensatory increase in expression in ECs to maintain cardiac function. However, eNOS/*NOS3* mRNA levels were significantly decreased in 14-week-old *db/db* mice, consistent with the impaired cardiac function at this stage (Fig. 7I). Taken together, these results reveal that miR-30e increases FAO and oxidative stress and impairs endothelial function in cultured ECs exposed to exogenous palmitate.

### Knock-down of the miR-30 family reduces oxidative stress and DNA damage in the coronary microvascular endothelium of diabetic mice

**Fig. 8:**
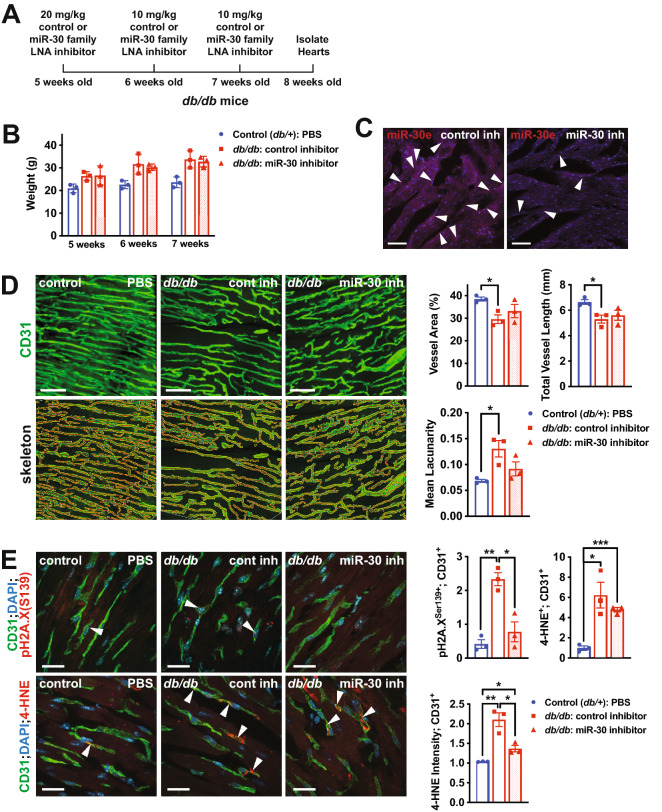
Inhibition of miR-30 decreases oxidative stress and DNA damage in microvascular endothelial cells in *db/db* mice. **A** Schematic of experimental set-up. Control or miR-30 family LNA inhibitor was injected intradermally in *db/db* mice at 5, 6 and 7 weeks of age and hearts were harvested at 8 weeks of age. Control (*db/* +) mice were injected with PBS at the same time-points. **B** Weights were measured at the time of injections, revealing normal weight gain in *db/db* mice injected with miR-30 inhibitor. **C** Confocal imaging of miRNAscope of miR-30e in left ventricles from *db/db* mice injected with control or miR-30 family LNA inhibitor. Arrowheads indicate expression in cells that are presumed to be endothelial cells based on morphology and anatomy. Representative images are shown. Scale bar = 50 μm. **D** 2-photon confocal microscopy of cardiac microvasculature in the left ventricle as assessed by CD31 immunofluorescence at 8 weeks of age in *db/db* mice (injected with control or miR-30 family LNA inhibitor) (right panels) and controls (*db/* +) (left panel). The top images are CD31 immunofluorescence and the bottom are the skeleton outlines of the microvasculature. Representative images are shown. Scale bar = 45 μm. Quantification of microvascular density, as assessed by measurement of microvascular area, total vessel length, and mean lacunarity (right). Each data point represents the mean of multiple fields of view from one mouse. * indicates p < 0.05 for the specified comparisons. **E** Representative images of immunofluorescent staining for pH2A.X (S139) and CD31 (top) and 4-HNE and CD31 (bottom) in the left ventricle in *db/db* mice (injected with control or miR-30 family LNA inhibitor) (right panels) and controls (*db/* +) (left panel) at 8 weeks of age. Scale bar = 22 μm. Arrowheads indicate examples of 4-HNE^+^ or pH2A.X^+^ ECs. Quantification of pH2A.X^+^;CD31^+^ double-positive ECs, 4-HNE^+^;CD31^+^ double-positive ECs and 4-HNE intensity in ECs is shown to the right. Each data point represents the mean of multiple fields of view from one mouse. *, ** and *** indicates p < 0.05, p < 0.01 or p < 0.001, respectively for the specified comparisons. All data in the figure depict mean ± SEM

To determine whether miR-30 up-regulation mediates microvascular dysfunction in vivo, we injected *db/db* mice with miR-30 family LNA inhibitor or control LNA inhibitor, at 5, 6 and 7 weeks of age and assessed vascular density and organization, oxidative stress (4-HNE) and DNA damage/senescence (phospho-H2A.X (Ser139), i.e. γH2A.X) at 8 weeks of age (Fig. 8A). There was no effect on weight gain in *db/db* mice injected with miR-30 family LNA inhibitor compared to control LNA inhibitor (Fig. 8B). Visualization of miR-30e by miRNAscope revealed a decrease in expression in the endothelium of mice injected with miR-30 family LNA inhibitor (Fig. 8C). Assessment of microvascular density revealed a trend towards improved vascular network coverage (i.e., an increase in vessel area and a reduction in lacunarity) in *db/db* mice with miR-30 knock-down, but this did not reach statistical significance (Fig. 8D). We also noted a significant decrease in the number of phospho-H2A.X^Ser139+^ (γH2A.X^+^) endothelial cells and a decrease in 4-HNE staining intensity in the microvascular endothelium in *db/db* mice injected with miR-30 family LNA inhibitor compared to control LNA inhibitor (Fig. 8E). Thus, miR-30 plays an important role in coronary microvascular dysfunction in diabetic mice by regulating oxidative stress and DNA damage/senescence in the endothelium.

## Discussion

### Summary of key findings

**Fig. 9:**
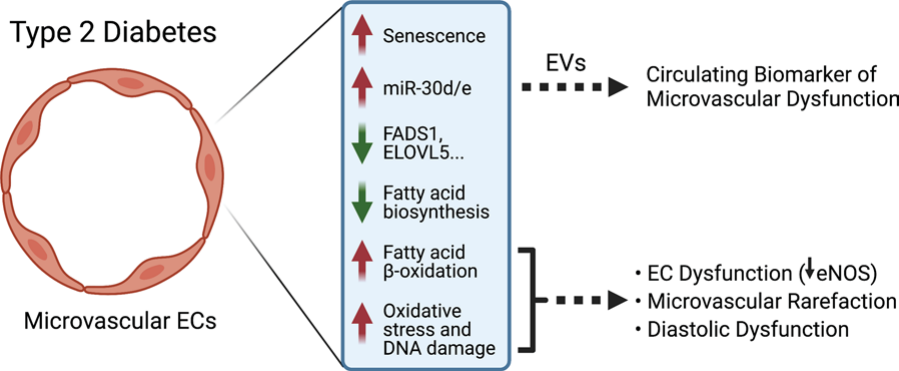
Schematic of the role of miR-30d/e in microvascular dysfunction in diabetes. Type 2 Diabetes (T2D) promotes microvascular dysfunction in the heart. In a mouse *db/db* model of T2D, microvascular rarefaction precedes echocardiographic evidence of diastolic dysfunction. The up-regulation of miR-30d/e in the endothelium of the heart is associated with induction of senescence-like pathways. MiR-30d/e can be secreted by the endothelium in extracellular vesicles (EVs) and detected in the circulation, and may serve as an early circulating biomarker of microvascular dysfunction. In the endothelium, miR-30d/e target a number of genes involved in fatty acid biosynthesis and in turn enhance β-oxidation of exogenous fatty acids. Over-expression of miR-30e results in oxidative stress, DNA damage and decreased levels of eNOS, which may contribute to microvascular dysfunction and rarefaction, ultimately contributing to diastolic dysfunction. The schematic was generated using Biorender

Here we observe that miR-30d and miR-30e are upregulated in circulating plasma EVs in obese and lean rodent models of T2D that display diastolic dysfunction. Importantly, we find that miR-30e (as well as several additional miRNAs) are up-regulated in circulating EVs at early stages of disease pathogenesis in *db/db* mice, coincident with coronary microvascular dysfunction and rarefaction, but preceding the development of diastolic dysfunction. Notably, miR-30d/e are up-regulated in the cardiac endothelium in *db/db* mice, but are not induced in other organs, suggesting that the circulating levels of these miRNAs may reflect, at least in part, their induction and secretion from dysfunctional cardiac microvasculature. We demonstrate that activation of senescence pathways in ECs leads to the production and secretion of miR-30d/e in EVs and that the coronary microvasculature has senescent-like characteristics. Furthermore, we discover that miR-30 regulates exogenous FAO in cultured ECs, promoting oxidative stress, lipid peroxidation and endothelial dysfunction (i.e., reduced eNOS expression) and that inhibition of the miR-30 family in vivo reduces markers of oxidative stress and DNA damage/senescence in the coronary microvasculature of *db/db* mice. Thus, the induction of miR-30d/e in cardiac endothelium may contribute to microvascular dysfunction and diastolic dysfunction in diabetes by enhancing FAO (See Fig. 9). Our findings reveal that miR-30d/e serve as potential biomarkers of diabetes-induced diastolic dysfunction in pre-clinical models, and this should be further assessed in human diabetic cohorts.

### miR-30 regulates FA metabolism in endothelial cells

Altered metabolism is implicated in T2D-associated cardiac dysfunction, but most prior work has focused on cardiomyocytes, where increased reliance on FAs as a fuel source is detrimental [46–48]. Less is known about whether metabolic changes in the endothelium contribute to cardiac dysfunction. Microvascular ECs transport FAs from the blood into the underlying parenchyma (including cardiomyocytes), but ECs can also transiently store FAs in lipid droplets to protect the cell from ER stress, and following lipolysis, FAO can be used as an energy source [40]. In ECs, FAO also contributes to the generation of dNTPs that are essential for proliferation [49] and is used to maintain redox homeostasis [50]. Our study reveals that miR-30 can promote lipid droplet formation and FAO in ECs. The miR-30-dependent increase in FAO may be detrimental, as this is associated with increased oxidative stress, lipid peroxidation, DNA damage and decreased eNOS expression. Augmented FAO in the endothelium may be especially damaging in the setting of T2D, where circulating triglyceride and FAs are highly elevated [15]. The enhancement in FAO could in part be due to the suppression of numerous genes that are involved in FA biosynthesis by miR-30d/e. This may in turn shift FA metabolic pathways towards storage and β-oxidation. This is consistent with a prior study that showed antagonism between FA biosynthesis and β-oxidation in the liver [51]. Our data shows that the miR-30 family plays a role in regulating how ECs handle FFA, potentially altering the metabolic response in the setting of T2D.

### The contribution of microvascular dysfunction to diastolic dysfunction

Similar to studies in T2D patients [12], we found that coronary microvascular dysfunction precedes the development of diastolic dysfunction. This suggests that targeting the decline in microvascular dysfunction is a potential therapeutic approach for diastolic dysfunction and HFpEF. As early as 8 weeks of age, *db/db* mice have coronary microvascular rarefaction, and the endothelium expresses markers of oxidative stress, DNA damage and senescence. Endothelial dysfunction and senescence accompany T2D and are likely the result of an overabundance of endothelium damaging factors in the blood stream, including pro-oxidant molecules, glucose and lipids or FFAs [52, 53]. Importantly, inducing senescence in ECs strongly up-regulates the expression and secretion of miR-30d/e. This is in line with previous studies that have shown miR-30 up-regulation during induced- and replicative-senescence [54]. In addition to the inflammatory pathways that are activated in senescent cells, our study suggests that induction of miR-30d/e in senescent ECs may also drive pathological FAO. Notably, previous studies have linked EC senescence to HFpEF-like phenotypes in a model of accelerated aging in mice [55] and removal of senescent cells improved diastolic function in an obese mouse model [56]. This suggests that targeting EC senescence may be a relevant therapeutic approach in T2D-associated cardiac dysfunction. Notably, inhibition of miR-30 in *db/db* mice reduced markers of oxidative stress, and DNA damage/senescence, and there was a trend towards improved microvascular network organization. This suggests that miR-30 may be functionally implicated in microvascular dysfunction that leads to diastolic dysfunction and HFpEF, potentially through regulation of FAO.

### Limitations and perspectives for future research

While our study has implicated miR-30 in the regulation of FA metabolism in ECs and coronary microvascular dysfunction in diabetes, there are several limitations of the current work. Our cell culture experiments utilized human umbilical vein endothelial cells (HUVECs) as a generic model of EC biology. Future studies should utilize cardiac microvascular ECs to determine whether there are any vascular bed-specific differences in miR-30-dependent pathways. Additionally, while our study focuses on the role of miR-30d/e within the endothelium, the implications for miR-30d/e-loaded EVs in cell–cell communication remains to be investigated.

Previous studies have revealed that circulating levels of miR-30 family members are increased in the early stages of T2D, and this miRNA family has been implicated in the regulation of diverse processes, including glucose metabolism, insulin signaling, inflammation and platelet activation (reviewed in Ref. [57]). Notably, the miR-30 family is one of the most abundantly expressed miRNAs in the heart [58]. Several contrasting studies have demonstrated that miR-30 family members participate in ventricular remodeling [58] and can have protective [59, 60] or detrimental [61] roles through regulation of multiple processes, including metabolism, pyroptosis, oxidative stress and autophagy. Of particular relevance, systemic inhibition of miR-30d in diabetic rats (high fat diet and streptozotocin model) can rescue systolic and diastolic function, potentially acting through regulation of autophagy in cardiomyocytes [62]. Furthermore, SGLT2 inhibition decreased miR-30d expression in this model, implying that miR-30 is detrimental in the setting of diabetes.

In contrast to these studies that have focused on cardiomyocytes, we found that miR-30d and miR-30e are induced in the endothelium of the heart in *db/db* mice and that over-expression of miR-30e in cultured ECs mediates elevations in FAO, oxidative stress and reduced eNOS expression. Furthermore, inhibition of miR-30 in *db/db* mice suppressed markers of oxidative stress and DNA damage/senescence in the microvasculature. Because of the diverse, and potentially opposing roles for miR-30 in different cell types in the heart, it may be necessary to develop endothelial-specific delivery modalities as a therapeutic approach. Importantly, while we have assessed the role of miR-30 in microvascular dysfunction at the early stages of T2D (i.e., 8 weeks of age), future studies will be needed to determine if prolonged inhibition can improve diastolic dysfunction (i.e., 14 weeks of age and beyond). EC-selective delivery approaches would be helpful for these studies.

Finally, it is worth noting that the pre-clinical diabetes models that we have used are genetic models where pathology develops relatively quickly. Future studies using a more physiological model of diabetes, such as diet-induced diabetes [63], will be informative to determine whether the mechanisms we have discovered are applicable to these models. Finally, it will be imperative to determine whether the miRNAs that we have identified could be utilized as early biomarkers of coronary microvascular dysfunction and diastolic dysfunction in human patients with T2D.

## Supplementary Information

**Additional file 1:** Data Supplement.

**Additional file 2: Table S1.** Primer sequences.

## Data Availability

All source data that support the findings of this study as well as any material used are available upon request from the corresponding author.

## References

[1] Defronzo RA (2009). Banting lecture. From the triumvirate to the ominous octet: a new paradigm for the treatment of type 2 diabetes mellitus. Diabetes.

[2] Kannel WB, Hjortland M, Castelli WP (1974). Role of diabetes in congestive heart failure: the Framingham study. Am J Cardiol.

[3] Kannel WB, McGee DL (1979). Diabetes and cardiovascular disease: the Framingham study. JAMA.

[4] Greenberg BH, Abraham WT, Albert NM, Chiswell K, Clare R, Stough WG (2007). Influence of diabetes on characteristics and outcomes in patients hospitalized with heart failure: a report from the Organized Program to Initiate Lifesaving Treatment in Hospitalized Patients with Heart Failure (OPTIMIZE-HF). Am Heart J.

[5] Bouthoorn S, Valstar GB, Gohar A, den Ruijter HM, Reitsma HB, Hoes AW (2018). The prevalence of left ventricular diastolic dysfunction and heart failure with preserved ejection fraction in men and women with type 2 diabetes: a systematic review and meta-analysis. Diabetes Vasc Dis Res.

[6] Arnold SV, Echouffo-Tcheugui JB, Lam CSP, Inzucchi SE, Tang F, McGuire DK (2018). Patterns of glucose-lowering medication use in patients with type 2 diabetes and heart failure. Insights from the Diabetes Collaborative Registry (DCR). Am Heart J.

[7] Patil VC, Patil HV, Shah KB, Vasani JD, Shetty P (2011). Diastolic dysfunction in asymptomatic type 2 diabetes mellitus with normal systolic function. J Cardiovasc Dis Res.

[8] Roh J, Houstis N, Rosenzweig A (2017). Why don't we have proven treatments for HFpEF?. Circ Res.

[9] Anker SD, Butler J, Filippatos G, Ferreira JP, Bocchi E, Bohm M (2021). Empagliflozin in heart failure with a preserved ejection fraction. N Engl J Med.

[10] Dryer K, Gajjar M, Narang N, Lee M, Paul J, Shah AP (2018). Coronary microvascular dysfunction in patients with heart failure with preserved ejection fraction. Am J Physiol Heart Circ Physiol.

[11] Mohammed SF, Hussain S, Mirzoyev SA, Edwards WD, Maleszewski JJ, Redfield MM (2015). Coronary microvascular rarefaction and myocardial fibrosis in heart failure with preserved ejection fraction. Circulation.

[12] Taqueti VR, Solomon SD, Shah AM, Desai AS, Groarke JD, Osborne MT (2018). Coronary microvascular dysfunction and future risk of heart failure with preserved ejection fraction. Eur Heart J.

[13] Riehle C, Bauersachs J (2018). Of mice and men: models and mechanisms of diabetic cardiomyopathy. Basic Res Cardiol.

[14] De Bock K, Georgiadou M, Schoors S, Kuchnio A, Wong BW, Cantelmo AR (2013). Role of PFKFB3-driven glycolysis in vessel sprouting. Cell.

[15] Sobczak IS, A, A Blindauer C, J Stewart A, (2019). Changes in plasma free fatty acids associated with type-2 diabetes. Nutrients.

[16] Wan A, Rodrigues B (2016). Endothelial cell-cardiomyocyte crosstalk in diabetic cardiomyopathy. Cardiovasc Res.

[17] Du X, Edelstein D, Obici S, Higham N, Zou MH, Brownlee M (2006). Insulin resistance reduces arterial prostacyclin synthase and eNOS activities by increasing endothelial fatty acid oxidation. J Clin Investig.

[18] Engin AB (2017). What Is lipotoxicity?. Adv Exp Med Biol.

[19] Ghosh A, Gao L, Thakur A, Siu PM, Lai CWK (2017). Role of free fatty acids in endothelial dysfunction. J Biomed Sci.

[20] Wang Y, Wang XJ, Zhao LM, Pang ZD, She G, Song Z (2019). Oxidative stress induced by palmitic acid modulates KCa2.3 channels in vascular endothelium. Exp Cell Res.

[21] Gustafson D, Veitch S, Fish JE (2017). Extracellular vesicles as protagonists of diabetic cardiovascular pathology. Front Cardiovasc Med.

[22] Colombo M, Raposo G, Thery C (2014). Biogenesis, secretion, and intercellular interactions of exosomes and other extracellular vesicles. Annu Rev Cell Dev Biol.

[23] van Niel G, D'Angelo G, Raposo G (2018). Shedding light on the cell biology of extracellular vesicles. Nat Rev Mol Cell Biol.

[24] Mulcahy LA, Pink RC, Carter DR (2014). Routes and mechanisms of extracellular vesicle uptake. J Extracell Vesicles.

[25] Njock MS, Cheng HS, Dang LT, Nazari-Jahantigh M, Lau AC, Boudreau E (2015). Endothelial cells suppress monocyte activation through secretion of extracellular vesicles containing antiinflammatory microRNAs. Blood.

[26] Horton WB, Barrett EJ (2021). Microvascular dysfunction in diabetes mellitus and cardiometabolic disease. Endocr Rev.

[27] Gu J, Wang S, Guo H, Tan Y, Liang Y, Feng A (2018). Inhibition of p53 prevents diabetic cardiomyopathy by preventing early-stage apoptosis and cell senescence, reduced glycolysis, and impaired angiogenesis. Cell Death Dis.

[28] Katsuumi G, Shimizu I, Yoshida Y, Minamino T (2018). Vascular senescence in cardiovascular and metabolic diseases. Front Cardiovasc Med.

[29] Shakeri H, Lemmens K, Gevaert AB, De Meyer GRY, Segers VFM (2018). Cellular senescence links aging and diabetes in cardiovascular disease. Am J Physiol Heart Circ Physiol.

[30] Shosha E, Xu Z, Narayanan SP, Lemtalsi T, Fouda AY, Rojas M (2018). Mechanisms of diabetes-induced endothelial cell senescence: role of arginase 1. Int J Mol Sci.

[31] Laudato S, Patil N, Abba ML, Leupold JH, Benner A, Gaiser T (2017). P53-induced miR-30e-5p inhibits colorectal cancer invasion and metastasis by targeting ITGA6 and ITGB1. Int J Cancer.

[32] Sohn D, Peters D, Piekorz RP, Budach W, Janicke RU (2016). miR-30e controls DNA damage-induced stress responses by modulating expression of the CDK inhibitor p21WAF1/CIP1 and caspase-3. Oncotarget.

[33] Yang H, Wang H, Ren J, Chen Q, Chen ZJ (2017). cGAS is essential for cellular senescence. Proc Natl Acad Sci USA.

[34] Flor AC, Kron SJ (2016). Lipid-derived reactive aldehydes link oxidative stress to cell senescence. Cell Death Dis.

[35] Yosef R, Pilpel N, Papismadov N, Gal H, Ovadya Y, Vadai E (2017). p21 maintains senescent cell viability under persistent DNA damage response by restraining JNK and caspase signaling. Embo J.

[36] Kuo LJ, Yang LX (2008). Gamma-H2AX - a novel biomarker for DNA double-strand breaks. In Vivo.

[37] Noren Hooten N, Evans MK. Techniques to induce and quantify cellular senescence. J Vis Exp. 2017;(123):55533. 10.3791/55533 PMC5565152 28518126

[38] Vlachos IS, Zagganas K, Paraskevopoulou MD, Georgakilas G, Karagkouni D, Vergoulis T (2015). DIANA-miRPath v3.0: deciphering microRNA function with experimental support. Nucleic Acids Res.

[39] Huang HY, Lin YC, Li J, Huang KY, Shrestha S, Hong HC (2020). miRTarBase 2020: updates to the experimentally validated microRNA-target interaction database. Nucleic Acids Res.

[40] Kuo A, Lee MY, Sessa WC (2017). Lipid droplet biogenesis and function in the endothelium. Circ Res.

[41] Lu Y, Qian L, Zhang Q, Chen B, Gui L, Huang D (2013). Palmitate induces apoptosis in mouse aortic endothelial cells and endothelial dysfunction in mice fed high-calorie and high-cholesterol diets. Life Sci.

[42] Hirota M, Kitagaki M, Itagaki H, Aiba S (2006). Quantitative measurement of spliced XBP1 mRNA as an indicator of endoplasmic reticulum stress. J Toxicol Sci.

[43] Ly LD, Xu S, Choi SK, Ha CM, Thoudam T, Cha SK (2017). Oxidative stress and calcium dysregulation by palmitate in type 2 diabetes. Exp Mol Med.

[44] Fleissner F, Thum T (2011). Critical role of the nitric oxide/reactive oxygen species balance in endothelial progenitor dysfunction. Antioxid Redox Signal.

[45] Forstermann U, Xia N, Li H (2017). Roles of vascular oxidative stress and nitric oxide in the pathogenesis of atherosclerosis. Circ Res.

[46] An D, Rodrigues B (2006). Role of changes in cardiac metabolism in development of diabetic cardiomyopathy. Am J Physiol Heart Circ Physiol.

[47] Isfort M, Stevens SC, Schaffer S, Jong CJ, Wold LE (2014). Metabolic dysfunction in diabetic cardiomyopathy. Heart Fail Rev.

[48] Stanley WC, Lopaschuk GD, McCormack JG (1997). Regulation of energy substrate metabolism in the diabetic heart. Cardiovasc Res.

[49] Schoors S, Bruning U, Missiaen R, Queiroz KC, Borgers G, Elia I (2015). Fatty acid carbon is essential for dNTP synthesis in endothelial cells. Nature.

[50] Kalucka J, Bierhansl L, Conchinha NV, Missiaen R, Elia I, Brüning U (2018). Quiescent endothelial cells upregulate fatty acid β-oxidation for vasculoprotection via redox homeostasis. Cell Metab.

[51] Huang H, McIntosh AL, Martin GG, Petrescu AD, Landrock KK, Landrock D (2013). Inhibitors of fatty acid synthesis induce PPAR alpha -regulated fatty acid beta -oxidative genes: synergistic roles of L-FABP and glucose. PPAR Res.

[52] Bonfigli AR, Spazzafumo L, Prattichizzo F, Bonafè M, Mensà E, Micolucci L (2016). Leukocyte telomere length and mortality risk in patients with type 2 diabetes. Oncotarget.

[53] Prattichizzo F, De Nigris V, Mancuso E, Spiga R, Giuliani A, Matacchione G (2018). Short-term sustained hyperglycaemia fosters an archetypal senescence-associated secretory phenotype in endothelial cells and macrophages. Redox Biol.

[54] Martinez I, Cazalla D, Almstead LL, Steitz JA, DiMaio D (2011). miR-29 and miR-30 regulate B-Myb expression during cellular senescence. Proc Natl Acad Sci USA.

[55] Gevaert AB, Shakeri H, Leloup AJ, Van Hove CE, De Meyer GRY, Vrints CJ (2017). Endothelial senescence contributes to heart failure with preserved ejection fraction in an aging mouse model. Circ Heart Fail.

[56] Palmer AK, Xu M, Zhu Y, Pirtskhalava T, Weivoda MM, Hachfeld CM (2019). Targeting senescent cells alleviates obesity-induced metabolic dysfunction. Aging cell.

[57] Pordzik J, Jakubik D, Jarosz-Popek J, Wicik Z, Eyileten C, De Rosa S (2019). Significance of circulating microRNAs in diabetes mellitus type 2 and platelet reactivity: bioinformatic analysis and review. Cardiovasc Diabetol.

[58] Zhang X, Dong S, Jia Q, Zhang A, Li Y, Zhu Y (2019). Biosci Rep..

[59] Yin Z, Zhao Y, He M, Li H, Fan J, Nie X (2019). MiR-30c/PGC-1beta protects against diabetic cardiomyopathy via PPARalpha. Cardiovasc Diabetol.

[60] Li J, Salvador AM, Li G, Valkov N, Ziegler O, Yeri A (2021). Mir-30d regulates cardiac remodeling by intracellular and paracrine signaling. Circ Res.

[61] Li X, Du N, Zhang Q, Li J, Chen X, Liu X (2014). MicroRNA-30d regulates cardiomyocyte pyroptosis by directly targeting foxo3a in diabetic cardiomyopathy. Cell Death Dis.

[62] Zhang WY, Wang J, Li AZ (2020). A study of the effects of SGLT-2 inhibitors on diabetic cardiomyopathy through miR-30d/KLF9/VEGFA pathway. Eur Rev Med Pharmacol Sci.

[63] Noyan-Ashraf MH, Shikatani EA, Schuiki I, Mukovozov I, Wu J, Li RK (2013). A glucagon-like peptide-1 analog reverses the molecular pathology and cardiac dysfunction of a mouse model of obesity. Circulation.

