# Supplementary material for: MiR-30 promotes fatty acid beta-oxidation and endothelial cell dysfunction and is a circulating biomarker of coronary microvascular dysfunction in pre-clinical models of diabetes
Source: Cardiovasc Diabetol. 2022 Feb 24;21:31. doi: 10.1186/s12933-022-01458-z (PMC8876371; doi:10.1186/s12933-022-01458-z)
Supplement: Supplementary file 1 — Additional file 1: Data Supplement. [file 12933_2022_1458_MOESM1_ESM.pdf]

MiR-30 promotes fatty acid beta-oxidation and endothelial cell dysfunction and is a circulating biomarker of coronary microvascular dysfunction in diabetes

Shawn Veitch, Makon-Sébastien Njock, Mark Chandy, M. Ahsan Siraj, Lijun Chi, HaoQi Li, Kai Yu, Kumaragurubaran Rathnakumar, Carmina Anjelica Perez Romero, Zhiqi Chen, Faisal J. Alibhai, Dakota Gustafson, Sneha Raju, Ruilin Wu, Dorrin Zarrin Khat, Yaxu Wang, Amalia Caballero, Patrick Meagher, Edward Lau, Lejla Pepic, Henry S. Cheng, Natalie J. Galant, Kathryn L. Howe, Ren-Ke Li, Kim A. Connelly, Mansoor Husain, Paul Delgado-Olguin, Jason E. Fish

Data Supplement:

Supplemental Methods:

Animal usage, housing and tissue harvesting – All animal protocols were approved by the Animal Care Committee at the University Health Network (Toronto) and St. Michaels Hospital (Toronto). Mouse and rat models of type 2 diabetes (T2D) were used in this study. Homozygous *Leptin-receptor* mutant, *Lepr*$^{db/db}$ (also known as *db/db*; B6.BKS(D)-*Lepr*^{db}/J; stock number 00697) male mice and age-matched non-diabetic heterozygote male controls (*db/*+) were purchased from Jackson Laboratories (Bar Harbour, ME), and non-obese, male diabetic Goto Kakizaki (GK) rats and non-diabetic male Wistar (WS) control rats were purchased from Charles River (Wilmington, MA). All animals were fed a normal chow diet. Fasting blood glucose levels were determined from tail venous blood with an automated glucose monitor (One Touch II; Lifescan, Inc., Milpitas CA). T2D mice were assessed primarily at 8 and 14 weeks of age, although some plasma and heart tissue samples were isolated at 4-6 weeks of age. T2D rats were assessed at 28 weeks of age. Multiple organs (left ventricle [LV] of the heart, brain, kidney, spleen, liver, skeletal muscle, visceral white adipose tissue) were harvested from *db/db* mice and controls at 14 weeks of age and were snap frozen for RNA isolation using Trizol (according to manufacturer's specifications). To collect RNA from circulating mononuclear cells, ~1 mL of blood was collected in EDTA tubes by cardiac puncture from anesthetized mice. RBCs were lysed by adding 20 mL of 1X RBC lysis buffer (Biolegend cat# 420301) to 1 mL of whole blood and vortexed, followed by incubation for 10 minutes (mins) at room temperature in the dark. Whole blood nucleated cells were pelleted by centrifugation at 350 g for 5 mins and the cell pellet was lysed in Trizol for RNA extraction.

In vivo miR-30 knock-down – Diabetic (*db/db*) mice were injected with miRCURY LNA miR-30 family power inhibitor (GeneGlobe ID: YFI0450026) or a control miRCURY LNA miRNA power inhibitor. Intradermal injections were performed at 5 weeks of age at 20 mg/kg, followed by injections at 6 and 7 weeks of age at 10 mg/kg. At 8 weeks of age, blood samples were collected in EDTA-coated tubes via cardiac puncture and hearts were harvested after perfusion with 5 mL of pre-chilled PBS and preserved in 4% paraformaldehyde for 8 hours at 4°C, following by dehydration with subsequent incubations in 10%, 20% and 30% sucrose for 18 hours each at 4°C. The hearts were then embedded in OCT (Tissue-Tek) and stored at -70°C until cryosectioning. Non-diabetic control (*db/*+) mice were included in the experiment and received PBS intradermal injections at the same time-points.

Preparation of palmitic acid and oleic acid solution and cell treatment – Palmitic acid or oleic acid solution was generated by conjugating palmitic acid (PA; Sigma-Aldrich; P5585) or oleic

acid (OA; Sigma-Aldrich; O1257) to methyl-β-cyclodextrin (MβCD; Sigma-Aldrich; C4555) in a 1:12 molar ratio to make an 8 mM PA-MβCD stock or a 5 mM OA-MβCD stock. Conjugation was mediated by dissolving PA or OA and methyl-β-cyclodextrin in sterile PBS and heating the solution to 60°C for 1 h with intermittent vortexing. The solution was then briefly sonicated over ice to ensure a homogeneous mixture. Samples were aliquoted to prevent freeze-thawing and stored at -20°C. For cell treatments, PA-MβCD or OA-MβCD was warmed to 37°C before addition to 1 mL of tissue culture media, making a final concentration of 40 μM, PA-MβCD or OA-MβCD. Treatment was performed for 24 h. For all cell culture treatments, an equivalent amount of methyl-β-cyclodextrin was added as a control at a final concentration of 280 μM.

Cell culture – Primary human umbilical vein endothelial cells (HUVEC) and media (Endothelial Cell Medium with 5% FBS and Endothelial Cell Growth Supplement) were purchased from ScienCell. Cells were used at passages 3–7 and were grown on tissue culture plastic coated with Attachment Factor (Gibco; S006100). To mimic diabetic conditions, HUVEC were exposed to tumor necrosis factor-α (TNF-α; 10 ng/ml; ThermoFisher; PHC3016), high glucose (25 mM), a combination of TNF-α (10 ng/ml) and high glucose (25 mM) or palmitate (40 μM) for 24 h. To model cellular senescence, cells were treated using two separate approaches. Irradiation experiments were performed using 10 Gy using a Cs-137 irradiator (Gammacell 40 Extractor – Best Theratronics). Control cells were placed outside of the incubator for an equal amount of time but not subjected to irradiation. Cells and extracellular vesicles (EVs) were collected 1 week after irradiation. Etoposide experiments were performed using 20 μM etoposide (Sigma; E1383) for 48 h, after which media was changed for 3 days before collection of cells and EVs. RNA was isolated from cells and reverse transcription and qPCR were performed (as described below) to assess expression of *CDKN2A* (p16) and *CDKN1A* (p21) and miR-30d and miR-30e. β-galactosidase was also assessed using the Senescence β-Galactosidase Staining Kit (Cell Signaling; 9860) following the manufacturer's recommended protocol. For EV isolation, media was changed to serum free media with EC growth factors for 24 h before isolation of EVs using the total exosome isolation reagent (ThermoFisher; 4478359), according to the manufacturer's recommendations.

Cell transfection – HUVEC were transfected (as done previously[1]) at 50-70% confluency with control or miR-30e mimic (40 nM final concentration, Dharmacon), given 24 h to recover before treatment, and were analyzed 24 h later. For inhibitor experiments, HUVEC were transfected at 80-90% confluency with control or miR-30 locked-nucleic acid (LNA) inhibitors (50nM, Power Inhibitors, Qiagen/Exiqon), given 48 h to recover before treatment, and analyzed 24 h later. All transfections were performed using Lipofectamine™ RNAiMAX (ThermoFisher; 13778150).

Extracellular vesicle isolation and characterization – To isolate circulating plasma EVs, total blood was isolated from animals using cardiac puncture and collected in EDTA-coated tubes. Platelet free plasma was collected using differential centrifugation at 1,500g for 10 minutes at 4°C to remove blood cells, and a second time at 3,000g for 20 minutes at 4°C to remove apoptotic bodies. 150 μL of plasma was used for extracellular vesicle (EV) isolation for downstream characterization or miRNA profiling. ExoQuick™ precipitation reagent (Systems Bioscience; EXOQ5A-1) was used according to the manufacturer's protocol. EV size and concentration was measured using NanoSight, NS300 (Malvern Instruments Ltd), nanoparticle tracking analysis (NTA) with the following parameters: camera level 14, temperature 25°C and three separate video recordings for 60 seconds (s). EV concentration was binned based on EV size, combining EVs

ranging in 25 nm size increments. Total EV concentration was calculated by averaging the concentration measured in the three technical measurements. Mode EV size was calculated by averaging the size mode from the three technical measurements. To isolate EVs from senescent HUVEC, cells were grown in 10 cm dishes coated with Attachment Factor (Gibco; S006100) and treated as discussed above. EVs were harvested using Total Exosome Isolation Reagent (ThermoFisher; 4478359) following manufacturer's recommended guidelines, at the same time as RNA was collected from cells using Trizol reagent.

Cryo-transmission electron microscopy (Cryo-TEM) – Cryo-TEM samples were prepared using Quantifoil R 2/2 Cu grids (#Q325CR2) negatively glow discharged in air. EV sample preparations (4 μL) were directly applied to grids and incubated in a Vitrobot (ThermoFisher Scientific) at 100% relative humidity (blot time 2.0 s, 4°C) before being plunge-frozen into liquid ethane. Vitrified samples were imaged using a Talos L120C transmission electron microscope (ThermoFisher Scientific) operating at 120 kV.

Echocardiographic assessment and strain analysis – Left ventricular (LV) function and strain analysis were analyzed using a Vevo 2100 system (VisualSonics) with a 30-MHz transducer (as before[2]). Images were acquired at greater than 200 frames/s. Mice were anesthetized with 1-2% isofluorane and placed in a supine position on an imaging handling platform which maintained the platform at 37°C with a heating system. The heart rate and respiratory rate were monitored via electrocardiogram (ECG) and respirometer. The transducer acquired M mode and B mode images in the parasternal long axis (PLA) and three orthogonal (mid-ventricular, apical and basal) parasternal short axis (PSA) orientations. Image analysis was performed by the echocardiographer using Vevo LAB (v3.1.1). The Vevo strain software suite was used to trace the endocardium and epicardium of the LV in the PLA orientation at end diastole. The Vevo strain integrated software tool calculated the longitudinal strain and strain rate from the PLA and the radial strain and strain rate were obtained from the PSA.

Hemodynamic measurements – In a terminal experiment, LV hemodynamics were measured immediately following echocardiography. A 1.4 F (mice) or 2 F (rats) conductance catheter (Millar Instruments, Inc, Houston, Tx) was inserted into the LV via a retrograde aortic approach. The maximal rates of ventricular pressure rise and fall (dp/dt_{max}, dp/dt_{min}) were calculated as indices of systolic and diastolic function, respectively. Ventricular relaxation was reflected by Tau, the time constant of mono-exponential decay of ventricular pressure during isovolumic relaxation, as previously published[2].

Analysis of microvascular networks – Hearts from *db/db* or control *db/+* mice were dissected in PBS, fixed in 4% PFA for 5 h, washed 3x in PBS and dehydrated in a methanol/PBS series for storage in 100% methanol at -20˚C. Samples were then rehydrated by reversing the methanol/PBS series, embedded in OCT (Tissue-Tek), and the blocks were frozen in isopentane. Hearts in OCT blocks were stored at -70° C until cryosectioning. 45 μm thick sections were permeabilized and blocked in PBS with 1.0% Triton X-100 and 5% heat-inactivated goat serum for 3 h at 4°C. Sections were then incubated with a 1:100 dilution of anti-Mouse CD31 antibody (BD Pharmingen, 553370) in PBS with 0.5% Triton X-100 and 5% heat-inactivated goat serum overnight at 4°C. Samples were washed 6 times, 1 h each with PBS with 0.5% Triton X-100 and 5% heat-inactivated goat serum, and then incubated overnight at 4°C with a 1:500 dilution of

secondary antibody (Alexa Fluor 488, Invitrogen, A-11029). Sections were washed again with PBS with 0.5% Triton X-100 and 5% heat-inactivated goat serum 6 times, 1 h each, and then with fresh solution overnight at 4°C. Sections were then mounted on glass slides with ProLong Gold Antifade Mountant (Thermo Fisher Scientific). Images across 26 μm Z-stacks were captured using a Leica SP8 Confocal with Two-Photon microscope. Stacks were used for quantification of vascular networks with AngioTool[3] or for generating three-dimensional renderings with Imaris Cell Imaging Software. Quantifications were averaged from three stacks per heart. For *GK* and Wistar control rats, 5 μm thick sections obtained from rat hearts embedded in paraffin were deparaffinized by washing in xylene 3 times 10 min each, followed by 2 washes 5 minutes each in absolute ethanol, once in 70% and once in 50% ethanol, before 2 washes 5 minutes each in PBS. Sections were then submerged in 0.01 M Sodium citrate pH 6.0 inside a pressure cooker, which was microwaved for three minutes after it reached full pressure (around 13 min). The pressure stopper was removed, and the cooker was put back in the microwave and heated for 7 more minutes. The slides in Sodium citrate were left to cool down to room temperature and washed 3 times 2 minutes each in PBS. The sections were then stained using the anti-CD31 antibody as above. CD31 positive signal was quantified using AngioTool on at least two sections from each animal.

Immunofluorescence – Heart sections were stained as described previously[4] and imaged using confocal microscopy. Primary antibodies used were as follows: anti-phospho-Histone H2A.X (Ser139) (203E; Cell Signaling Technology #9718, 1:100 dilution); anti-4-Hydroxynonenal (4-HNE) (Abcam ab46545, 1:100 dilution); anti-mouse CD31 (BD Pharmingen 553370, 1:100 dilution). Secondary antibodies used were as follows: goat anti-mouse Alexa Fluor 488 (Thermo Fisher Scientific A-11029, 1:500 dilution); goat anti-rabbit Alexa Fluor 594 (Thermo Fisher Scientific A-11012, 1:500 dilution). Cells double positive for CD31 and phospho-Histone H2A.X or 4-HNE per view field were quantified from at least three view fields per section, in three sections from each heart analyzed.

Tissue and cell mRNA gene expression analysis – RNA was isolated using Trizol (Invitrogen) following the manufacturer's recommended protocol. Reverse transcription was performed with the High Capacity Reverse Transcription kit (Applied Biosystems; 4368814) using a 10 μL reaction mixture containing 3.2 μL nuclease-free water, 2 μL 10x Reverse Transcriptase Buffer, 2 μL Reverse Transcriptase Random Primers, 1 μL RNase Inhibitor, and 1 μL MultiScribe™ Reverse Transcriptase 50 U/μL. An input of 1 μg of total RNA, in a final volume of 10 μL, was added, and incubated as follows: 25°C for 10 min, 37°C for 120 min, 85°C for 5 min, and then immediately cooled to 4°C. Final cDNA product was diluted 1:4 in nuclease-free water and then stored at -20°C until further use. Quantitative reverse-transcriptase PCR (qRT-PCR) was performed as described previously[5]. qRT-PCR was conducted in triplicate using a Roche Lightcycler 480® and Roche LC 480 SYBR Green I Master (Roche; 04707516001) following the manufacturer's recommended protocol. A 5 μL reaction mixture was used containing 3.0 μL Sybr Green Master Mix, 1.5 μL nuclease free water, 0.25 μL 10 μM forward primer, and 0.25 μL 10 μM reverse primer. Briefly, the qRT-PCR protocol was as follows, using a final reaction volume of 6 μL with 5 μL of reaction mix and 1 μL of cDNA: Pre-incubation 1 cycle at 95°C for 5 min; amplification 45 cycles at 95°C for 10 sec, 60°C for 10 sec, 72°C for 8 sec; melting curve at 95°C for 5 sec, 65°C for 1 min, and 95°C continuously; finally cooling at 40°C for 10 sec. Data was normalized to Tata box binding protein (TBP) or glyceraldehyde 3-phosphate dehydrogenase

(GAPDH) and was analyzed using the Delta-Delta C_t method[6]. The primers used are indicated in Additional File 1: Table S1.

Tissue and cell microRNA gene expression analysis – RNA was isolated using Trizol (ThermoFisher; 15596026) following the manufacturer's recommended protocol. Reverse transcription was performed with the miScript II RT kit (Qiagen; 218161) using a 10 µL reaction mixture containing 4 µL 5x miScript HiSpec Buffer, 2 µL 10x miScript Nucleics Mix, 2 µL nuclease-free water, and 2 µL miScript Reverse Transcriptase Mix. An input of 100 ng of total RNA, in a final volume of 10 µL, was added and incubated as follows: 37°C for 60 min, 95°C for 5 min, then cooled to 4°C. Final cDNA product was diluted 1:4 nuclease-free water and then stored at -20°C until further use. qRT-PCR was performed using the miScript SYBR Green PCR Kit (Qiagen; 218073). qRT-PCR was conducted in triplicate using a Roche Lightcycler 480® using a reaction volume of 8 µL containing 5 µL 2x QuantiTect SYBR Green PCR Master Mix, 1 µL 10x miScript Universal Primer, 1.4 µL nuclease-free water, and 0.6 µL 10 µM miScript primer assay. Briefly, the qRT-PCR protocol was as follows, using a final reaction volume of 10 µL with 8 µL of reaction mix and 2 µL of cDNA: Preincubation 1 cycle at 95°C for 15 min; amplification 50 cycles at 94°C for 15 sec, 55°C for 30 sec, and 70°C for 30 sec. Data was normalized to small nucleolar RNA-U6 (snoU6) and was analyzed using the Delta-Delta C_t method[6]. The primers used are indicated in Additional File 1: Table S1.

EV-microRNA array/microfluidics and gene expression analysis – Circulating plasma EV-microRNAs (miRNA) were measured in mouse (*db/db*) and rat (GK) models of type 2 diabetes using two distinct miRNA platforms; a qRT-PCR miRNA array as well as a microfluidics miRNA array platform to detect the expression of miRNAs and multiple small RNA normalization controls. EVs were purified (as detailed above) from 150 µl of plasma using ExoQuick™.

For the qRT-PCR miRNA array, RNA was isolated using SeraMir exosome RNA purification kit (System Biosciences; RA808A-1) following the manufacturer's recommended protocol. Reverse transcription was performed (as detailed above) using 10 ng of total RNA with the miScript II RT Kit (Qiagen; 218161), and miRNA expression was detected using mouse or rat miScript miRNA PCR Serum and Plasma Arrays (Qiagen; 331221; GeneGlobe ID MIMM-106Z) to measure a panel of 84 miRNAs and small RNA controls (as detailed above) with a Roche Lightcycler 480®. Mouse miRNA PCR array data was normalized to the average of four separate house-keeping genes; miR-222, miR-191, Let-7a, miR-26a. Rat miRNA PCR array data was normalized to the average of two separate house-keeping genes; miR-191 and Let-7a. House-keeping genes were identified using GeNorm software[8] and the data was analyzed using the Delta-Delta C_t method. To compare expression levels between groups, unpaired student's t-tests were utilized. The panel of miRNAs assessed are outlined in Additional File 1: Table S1.

For the microfluidics miRNA array, RNA was isolated with the SeraMir exosome RNA purification kit (System Biosciences; RA808A-1) following the manufacturer's recommended protocol. Reverse transcription was carried out with the Universal cDNA synthesis kit II (Qiagen; 339340) using a 16 µl reaction mixture containing 4 µL 5x reaction buffer, 9 µL nuclease-free water, 2 µL enzyme mix, and 1 µL synthetic U6 and Cel-miR-39 spike-in. An input of 20 ng of total RNA (5 ng/µL) was added to the reaction mixture and incubated as follows: 42°C for 60 min, 95°C for 5 min, and then immediately cooled to 4°C. Final cDNA products were diluted 1:10 in nuclease-free water and stored at -20°C until downstream amplification. Pre-amplification of cDNA was facilitated by creating a pool of 96 LNA™ miRNA Assays (Qiagen; 339306) at a final

concentration of 0.25X for each assay. The pre-PCR amplification reaction was performed in a 5 µl reaction mixture containing 2.5 µl TaqMan PreAmp Master Mix 2X (Applied Biosystems; 4391128), 1.25 µl of 96-pooled LNA™ assay mix (0.25X), and 1.25 µl of diluted cDNA. The pre-amplification PCR was performed according to the following cycling conditions: one cycle 95°C for 10 minutes, 15 cycles at 95°C for 15 seconds, and then 60°C for 4 minutes. After pre-amplification PCR, the product was then diluted 1:10 in nuclease-free water and stored at -80°C until needed for amplification. qRT-PCR of the miRNA targets was carried out using the 96.96 dynamic array (Fluidigm) following Qiagen's recommended protocol. Briefly, a pre-sample mixture was prepared for each sample containing 275 µL of 2X TaqMan® Gene Expression Master Mix (Applied Biosystems; 4369542), 27.5 µL of 20X DNA Binding Dye Sample Loading Reagent (Fluidigm Corporation; 100-7609) 27.5 µL of EvaGreen™ (Biotium; 31000), and 82.5 µL 1X TE Buffer. 3.75 µL pre-sample mix was then combined with 1.25 µL each of diluted pre-amplified cDNA. Five microliters of unique assay mixes were prepared by combining 1.25 µL of a 1X LNA™ miRNA assay, 2.5 µL of 2X Assay Loading Reagent (Fluidigm Corporation; 100-7611), and 1.25 µL 1X TE Buffer. Dynamic Arrays (Fluidigm Corporation; BMK-M-96.96) were primed with control line fluid in the IFC controller and 5 µL of both sample and assay mixes loaded into the appropriate inlets. The chip was then loaded as well as mixed in the IFC controller, and then placed in the BioMark HD Instrument (Fluidigm Corporation) for PCR at 95°C for 10 minutes, followed by 35 cycles at 95°C for 10 seconds and 60°C for 1 minute. Raw data were analyzed and exported with Real-Time PCR Analysis Software (Fluidigm Corporation); data were normalized to the global mean expression of all miRNAs. GenEx 6 software (MultiD, Gothenbrug, Sweden) was used for data pre-processing, cleaning, and transformation. Raw Cq values were transformed to relative quantities and expression values were converted to log_2 scale. To compare expression levels between groups, unpaired student's t-tests were utilized. It is important to note that of the 96 primer sequences on the Exiqon LNA microfluidics qPCR panel (Additional File 1: Table S1), 58 were the same as the Qiagen miRNA array. All of the miRNAs that we found to be differentially regulated using the Qiagen miRNA array were also present on the Exiqon microfluidics platform.

Validation of individual circulating plasma EV-miRNA was performed using standard qRT-PCR. EVs were purified (as detailed above) from 150 µL of plasma using ExoQuick™. RNA was isolated using SeraMir (System Biosciences) following the manufacturer's recommended protocol. Reverse transcription was performed with the miScript II RT Kit (Qiagen; 218161) using 10 ng of total RNA (as detailed above). MiRNA expression was detected (as detailed above) using the miScript SYBR Green PCR Kit (Qiagen; 218073). qRT-PCR was conducted in triplicate using a Roche Lightcycler 480®. Data was normalized to the average of four separate house-keeping genes; miR-222, miR-191, Let-7a, and miR-26a (for time course validation), or miR-191 alone (for individual miRNA validation), and was analyzed using the Delta-Delta C_t method.

Investigation of tissue culture EV-miRNA expression was performed using standard qRT-PCR. EVs were purified (as detailed above) from tissue culture media using Total Exosome Isolation Reagent (ThermoFisher; 4478359). RNA was isolated using miRNeasy Micro Kit (Qiagen; 217084) following the manufacturer's recommended protocol. Reverse transcription was performed using the miScript II RT Kit (Qiagen; 218161) using 10 ng of total RNA (as detailed above). MiRNA expression was detected (as detailed above) using the miScript SYBR Green PCR Kit (Qiagen; 218073). qRT-PCR was conducted in triplicate using a Roche Lightcycler 480®. Data was normalized to miR-191 and was analyzed using the Delta-Delta C_t method.

CD31⁺ EC isolation – Left ventricles (LV) from 14-week-old *db/db* mice and *db/+* controls were dissected and placed in cold PBS to remove residual blood from the heart. The heart was placed in a 60 mm dish and 500 μL of a dissociation buffer, consisting of Collagenase IV (200 units/ml), 1:100 chicken serum and 2.5% of trypsin, was added. The heart was minced using a sterile razor blade, and an additional 1.5 ml of dissociation buffer was added to the minced LV. The minced heart was equally divided into two cryotubes, and the tubes were rotated inside a 5% CO_2 incubator at 37°C for 10 min. The cell suspension was pipetted up and down 3 times, and once settled the supernatant was collected and 250 μL/mL of horse serum was added. The digestion was repeated four times using 2 mL of fresh digestion buffer each time. Once complete, the cells were filtered using a 70 μm nylon filter, centrifuged at 200 g for 5 min, and three groups of cells were prepared: Unstained, DAPI alone to detect dead cells, or anti-CD31-Phycoerythrin (PE) rat antibody (Biolegend; 102407). Cells were stained with Anti-CD31-PE for 30 mins at room temperature before staining with DAPI. Cells were washed in sorting buffer and spun again at 200g for 5 minutes at 4°C. Pellets were resuspended and PE^+ cells were sorted by flow activated cell sorting using the FACSAria™ Fusion Cell Sorter, collecting approximately 250,000 cells from each group. RNA was isolated from PE^+ and PE^- fractions using the miRNeasy Micro Kit (as described previously), reverse transcriptase cDNA synthesis was performed using the High Capacity Reverse Transcription Kit for mRNA (as described previously) and miRNA using the miScript II RT Kit (as described previously). qRT-PCR was performed using the Roche LC 480 SYBR Green I Master Mix for mRNA (as described previously) and the miScript SYBR Green PCR Kit for miRNA (as described previously). qRT-PCR was conducted in triplicate using a Roche Lightcycler 480®. Data was normalized to TBP for mRNA and snoU6 for miRNA, and was analyzed using the Delta-Delta C_t method.

miRNAscope analysis of miR-30e-5p expression – miRNAscope was performed according to the manufacturer's recommendations using the miRNAscope HD (RED) Assay (324510-UM) (Advanced Cell Diagnostics [ACD], Newark, CA). The SR-mmumiR-30e-5p-S1 probe (Cat.#:1129241-S1) was designed by ACD (Newark, CA). Importantly, some cross-hybridization with other miR-30 family members is possible because of the similarity in sequence. Hearts from *db/db* or control *db/+* mice were dissected in PBS, fixed in 4% PFA for 5-8 h, washed 3x in PBS and dehydrated in a methanol/PBS series for storage in 100% methanol at -20˚C. Samples were then rehydrated by reversing the methanol/PBS series, embedded in OCT (Tissue-Tek), and the blocks were frozen in isopentane. Hearts in OCT blocks were stored at -70°C until cryosectioning at 20 μm. On day one of the staining protocol, slides with fixed frozen tissue sections were baked for 60 min at 60°C, followed by 15 min incubation in pre-chilled 10% neutral buffered formalin (NBF) at 4°C. After fixation, tissue sections were dehydrated with subsequent incubations in 50%, 70%, 100% (2x) ethanol for 5 min each at room temperature. The sections were re-immersed in fresh 10% NBF for post fixation over-night at room temperature. On day 2, the slides were washed in water and air dried at 60°C for 5 min. 2–4 drops of RNAscope Hydrogen Peroxide was added to each section for 10 min at room temperature. For target retrieval, 1X target retrieval buffer (Ref 322000; ACD, Newark, CA) and distilled water were heated to above 99°C. Slides were dipped in water for 10 seconds before placing into the 1X target retrieval buffer for 5 min. Then, the hot slide rack was transferred to a staining dish containing distilled water. The slides were washed in fresh distilled water 2 times and in 1xPBS one time. The slides were air dried at 60°C for 5 min. A hydrophobic barrier was drawn around the sections and RNAscope Protease III (Ref 322340; ACD, Newark, CA) was added to cover each section and incubated at 40°C for 20 min inside the

RNAscope HybEZ II Oven. For probe hybridization, 1X probe mixture was added to each section and incubated at 40°C for 2 h in the RNAscope HybEZ II Oven. For Amp 1-6 hybridization, 1X Amp 1 solution was added to each section and incubated at 40°C for 30 min in the RNAscope HybEZ II Oven according to the user manual. Amp hybridization steps were sequentially repeated with Amp 2-6 solutions. For detecting signal and counterstaining, 150-200 µL of working red solution was added per slide using a 1:60 ratio of Red-B to Red-A and incubated for 10 min at room temperature. DAPI (Ref 320858; ACD, Newark, CA) was added to each slide for 10 min at room temperature before being replaced with ProLong Gold Antifade Mountant (Ref P36930; Invitrogen, Waltham, MA). Necessary wash steps with the appropriate buffers were done in between each step above as indicated in the User Manual. Controls were performed in which no probe was added, but all other steps were performed identically. Slides were imaged with an Olympus FluoView FV1000 Laser Scanning Confocal Microscope Olympus IX81 inverted stand; 40X objective lens Plan Apo 40x/1.35 NA oil immersion.

Seahorse extracellular flux analysis – Cellular fatty acid β-oxidation (FAO) was measured using a Seahorse XFe24 extracellular flux analyzer (Agilent Technologies, CA, USA) in intact HUVEC as detailed previously[9]. Cells were transfected with control or miR-30e mimic (40 nM) or control or miR-30 power LNA inhibitor (50 nM) for 4 h and given 24 h (for mimic) or 48 h (for inhibitor) to recover (as described above). Cells were then plated on a 24-well seahorse V7 plate and cultured in growth media (ScienCell Endothelial Cell Growth Media) for 24 h, followed by overnight starvation in substrate-limited media (DMEM, 0.5 mM D-Glucose, 1 mM GlutaMAX, 0.5 mM Carnitine and 1% FBS). Cells were then incubated in FAO assay media (Seahorse base media, 2.5 mM D-Glucose, 0.5 mM Carnitine and 5 mM HEPES) at 37°C in an incubator without CO_2 for 45 min, followed by the addition of etomoxir (40 µM) and XF palmitate-BSA FAO substrate (200 µM) or XF BSA-control (Agilent#102720-100) to the FAO media immediately prior to the assay. Mito stress test (Agilent #102340-100) was performed by sequential addition of Oligomycin (1 µM), FCCP (1 µM), Antimycin-A (2 µM) and Rotenone (2 µM). Exogenous palmitate oxidation was calculated by assessing maximum oxygen consumption rate (OCR) after FCCP injection minus minimum OCR after AntA/rotenone and normalized to total cellular protein concentrations, as per manufacturer's instructions.

Measurement of Reactive Oxygen Species – HUVECs were transfected with control or miR-30e mimic (40 nM) for 4 h and given 24 h to recover (as described above). After 24 h, 40,000 cells per well were transferred to a 96 well black walled plate, and cells were grown overnight. The following day, the media was changed to DMEM without glucose (GIBCO), containing 0.5 mM D-Glucose, 1 mM GlutaMAX, 0.5 mM Carnitine and 1% FBS (as done for Seahorse assays) and cells were treated with 40 µM of palmitic acid for 1 h or 4 h. Following treatment, cells were washed 2x with PBS and then treated with 5 µM CellRox Green (ThermoFisher) for 1 h. Following 3x washes with PBS, fluorescence was measured using the Cytation™ 5 Cell Imaging Multi-Mode Reader (BioTeK Instruments, Inc.) at an excitation/emission spectra of 485/520 nm to detect ROS. Experiments were performed with technical triplicates and was analyzed by subtracting the fluorescence from background.

Measurement of 4-HNE in cultured cells – HUVECs were seeded on 12 well plates coated with Attachment Factor (Gibco, S-006-100) at a density of 250,000 cells per well. The following day the cells were starved for an hour in OpiMEM medium (Gibco, 31985-070), then transfected with

control or miR-30e mimic (40 nM final concentration, Dharmacon) using Lipofectamine™ RNAiMAX (ThermoFisher; 13778150) for 4 hours and given 24 hours to recover. After 24 hours, cells were treated with 40 μM of palmitic acid (in Methyl-β-cyclodextrin) or an equal volume of Methyl-β-cyclodextrin for 1 hour. After treatment, cells were washed with 1X PBS twice before harvesting in RIPA lysis buffer. The concentration of 4-HNE protein in the lysate was assessed by the Lipid Peroxidation (4-HNE) Assay Kit (Abcam, ab238538) using a standard curve and quantified by the Cytation™ 5 Cell Imaging Multi-Mode Reader (BioTeK Instruments, Inc.) at an absorption of 450 nm with background subtraction.

BODIPY staining of lipid droplets – HUVEC were transfected with negative control mimic or miR-30e mimic (as described above). After 24 h, 80,000 cells per well were transferred to an 8-well chamber slide coverslips (Ibidi) coated with 1 mg/mL fibronectin, and cells were grown overnight. The following day, cells were treated with 40 μM of palmitic acid or 40 μM or oleic acid or an equal volume of methyl-β-cyclodextrin for 24 h. Following treatment, cells were washed 3x with $PBS^{-/-}$ and were fixed with 4% paraformaldehyde in $PBS^{-/-}$ for 20 minutes at room temperature. Following fixation, cells were washed 3x with $PBS^{-/-}$, and stained with BODIPY 493/503 (ThermoFisher; D3922) diluted in $PBS^{-/-}$ at a final concentration of 0.1 mg/mL for 15 min at room temperature to delineate lipid droplets. Cells were washed 3x with $PBS^{-/-}$ and coverslips were mounted with Vectashield Mounting Medium with DAPI (Vector Lab Inc.; H-1200) and imaged by laser-scanning confocal microscopy (Olympus Fluoview 1000) with Plan Apo 40x/1.35 NA oil immersion objective lens at room temperature using a 405 nm laser for DAPI and 473 nm laser for AlexaFluor488. Images were processed using the FV10-ASW 4.2 Viewer and fluorescence was quantified using ImageJ software. Analysis was performed on 4 individual cells from 3 fields of view, from 2 separate biological replicates, and background fluorescence was subtracted from values.

Western blotting – Western blots were performed as before[10-12]. For miR-30 targets and endothelial markers, 10-20 μg of protein was loaded and the antibodies used were; FADS1 (Abcam; ab240024; 1:1000), ELOVL5 (Abcam; ab205535; 1:1000), eNOS (Santa Cruz, C-20; 1:1000) and β-actin (Cell Signaling; 4967s; 1:500). For EV quality control markers, 10 μg of protein was loaded (with the exception of CD63 where maximal volume was loaded in the well), and the antibodies used were; CD81 (Rabbit polyclonal; ABclonal; A5270; 1:1000), CD63 (Rabbit polyclonal; ABclonal; A5271; 1:500), TSG101 (Rabbit polyclonal; ABclonal; A2216; 1:1000) and Calnexin (Rabbit polyclonal; ABclonal; A15641; 1:1000). Densitometry was performed using the gel analysis tool in ImageJ. Densitometry values were normalized to the house-keeping gene (i.e., β-actin).

Informatics – KEGG pathway analysis of potential miR-30d-5p and miR-30e-5p target genes was performed using prediction software (DIANA-miRPath v3.0 with Tarbase v7.0)[13]. Separate analysis was performed for human and mouse.

Statistical analysis – All experiments were performed at least 3 independent times on biological replicates unless otherwise stated. The exact number of replicates is stated in the figure legends or indicated in the figures as individual data points. Data plots depict mean +/- standard error of the mean unless stated otherwise. Statistical analysis was performed in GraphPad Prism (version 9.2.0) using an unpaired two-sided Student's t-test for pairwise comparisons, and One-Way ANOVA

with Holm-Sidak's multiple comparisons test for multiple comparisons, unless stated otherwise. For all figures, *, ** and *** indicate $p<0.05$, $p<0.01$ and $p<0.001$, respectively.

References:

1. Cheng HS, Sivachandran N, Lau A, Boudreau E, Zhao JL, Baltimore D, Delgado-Olguin P, Cybulsky MI and Fish JE. MicroRNA-146 represses endothelial activation by inhibiting pro-inflammatory pathways. *EMBO Mol Med*. 2013;5:1017-34.
2. Bugyei-Twum A, Ford C, Civitarese R, Seegobin J, Advani SL, Desjardins JF, Kabir G, Zhang Y, Mitchell M, Switzer J, Thai K, Shen V, Abadeh A, Singh KK, Billia F, Advani A, Gilbert RE and Connelly KA. Sirtuin 1 activation attenuates cardiac fibrosis in a rodent pressure overload model by modifying Smad2/3 transactivation. *Cardiovascular research*. 2018;114:1629-1641.
3. Zudaire E, Gambardella L, Kurcz C and Vermeren S. A computational tool for quantitative analysis of vascular networks. *PloS one*. 2011;6:e27385.
4. Roy AR, Ahmed A, DiStefano PV, Chi L, Khyzha N, Galjart N, Wilson MD, Fish JE and Delgado-Olguin P. The transcriptional regulator CCCTC-binding factor limits oxidative stress in endothelial cells. *The Journal of biological chemistry*. 2018;293:8449-8461.
5. Fish JE, Yan MS, Matouk CC, St Bernard R, Ho JJ, Gavryushova A, Srivastava D and Marsden PA. Hypoxic repression of endothelial nitric-oxide synthase transcription is coupled with eviction of promoter histones. *The Journal of biological chemistry*. 2010;285:810-26.
6. Livak KJ and Schmittgen TD. Analysis of relative gene expression data using real-time quantitative PCR and the 2(-Delta Delta C(T)) Method. *Methods (San Diego, Calif)*. 2001;25:402-8.
7. Matin F, Jeet V, Moya L, Selth LA, Chambers S, Clements JA and Batra J. A Plasma Biomarker Panel of Four MicroRNAs for the Diagnosis of Prostate Cancer. *Scientific reports*. 2018;8:6653.
8. Vandesompele J, De Preter K, Pattyn F, Poppe B, Van Roy N, De Paepe A and Speleman F. Accurate normalization of real-time quantitative RT-PCR data by geometric averaging of multiple internal control genes. *Genome Biol*. 2002;3:RESEARCH0034.
9. Siraj MA, Mundil D, Beca S, Momen A, Shikatani EA, Afroze T, Sun X, Liu Y, Ghaffari S, Lee W, Wheeler MB, Keller G, Backx P and Husain M. Cardioprotective GLP-1 metabolite prevents ischemic cardiac injury by inhibiting mitochondrial trifunctional protein-alpha. *The Journal of clinical investigation*. 2020;130:1392-1404.
10. Fish JE, Cantu Gutierrez M, Dang LT, Khyzha N, Chen Z, Veitch S, Cheng HS, Khor M, Antounians L, Njock MS, Boudreau E, Herman AM, Rhyner AM, Ruiz OE, Eisenhoffer GT, Medina-Rivera A, Wilson MD and Wythe JD. Dynamic regulation of VEGF-inducible genes by an ERK/ERG/p300 transcriptional network. *Development (Cambridge, England)*. 2017;144:2428-2444.
11. Fish JE, Santoro MM, Morton SU, Yu S, Yeh RF, Wythe JD, Ivey KN, Bruneau BG, Stainier DY and Srivastava D. miR-126 regulates angiogenic signaling and vascular integrity. *Developmental cell*. 2008;15:272-84.
12. Fish JE, Wythe JD, Xiao T, Bruneau BG, Stainier DY, Srivastava D and Woo S. A Slit/miR-218/Robo regulatory loop is required during heart tube formation in zebrafish. *Development (Cambridge, England)*. 2011;138:1409-19.
13. Vlachos IS, Zagganas K, Paraskevopoulou MD, Georgakilas G, Karagkouni D, Vergoulis T, Dalamagas T and Hatzigeorgiou AG. DIANA-miRPath v3.0: deciphering microRNA function with experimental support. *Nucleic Acids Res*. 2015;43:W460-6.

Supplemental Figure Legends:

Figure S1: Cardiac functional parameters in Goto-Kakizaki (GK) diabetic rats and *db/db* diabetic mice. **(A)** Multiple select parameters (heart rate, fractional shortening, left ventricular (LV) mass, LV diastolic volume) in *db/db* mice and controls at 14 weeks reveal normal systolic function. **(B)** Fasting blood glucose levels and body weight in GK rats and Wistar controls at 28 weeks of age. **(C)** Longitudinal and radial strain analysis of the parasternal long axis of GK rats and Wistar controls at 28 weeks. **(D)** Pressure-Volume (PV) loop analysis of GK rats and Wistar controls at 28 weeks. **(E)** Tau logistic from PV loop analysis of GK rats and Wistar controls at 28 weeks. **(F)** Multiple select parameters (heart rate, ejection fraction, fractional shortening, left ventricular (LV) mass, LV volume during diastole) were measured by echocardiography at 28 weeks in GK rats and Wistar controls. All analyses in this figure were performed using unpaired t-tests. *, ** and *** indicate $p<0.05$, $p<0.01$ and $p<0.001$, respectively, for the specified comparisons. NS = not significant. All data in the figure depict mean +/- SEM.

Figure S2: GK diabetic rats have coronary microvascular rarefaction. **(A)** 2-photon confocal microscopy of cardiac microvasculature in the left ventricle as assessed by CD31 immunofluorescence at 28 weeks in Goto-Kakizaki (GK) rats and Wistar controls. The top images are CD31 immunofluorescence with DAPI staining and the bottom are the skeleton outlines of the microvasculature. Representative images are shown. Scale bar = 50 µm. **(B)** Quantification of microvascular density, as assessed by measurement of microvascular area and mean lacunarity in 28-week *GK* and Wistar control rats. Each data point represents the mean of multiple fields of view from one rat. * and ** indicate $p<0.05$ and $p<0.01$, respectively, for the specified comparisons using an unpaired t-test. All data in the figure depict mean +/- SEM.

Figure S3: Extracellular vesicle size and concentration are increased in GK rats. **(A)** Nanoparticle tracking analysis of plasma EVs from Goto-Kakizaki (GK) and Wistar (WS) control rats at 28 weeks of age, binned by size. n=4. **(B)** Quantification of EV concentration across all size bins and mode particle size from GK and WS controls at 28 weeks. * and ** indicate $p<0.05$ and $p<0.01$, respectively, for the specified comparisons using an unpaired t-test. All data in the figure depict mean +/- SEM.

Figure S4: Differential miRNA levels in circulating EVs isolated from *db/db* mice and GK rats. **(A)** Individual data points for significantly dysregulated miRNAs in *db/db* vs. control EVs at 14 weeks as determined by qRT-PCR array (using an unpaired t-test). Heatmap summarizing this data is shown in Figure 3A. **(B)** Individual data points for significantly dysregulated miRNAs in *db/db* vs. control EVs at 14 weeks as determined by microfluidics (using an unpaired t-test). Data is log2 transformed. Heatmap summarizing this data is shown in Figure 3B. **(C)** Individual data points for significantly dysregulated miRNAs in GK vs. Wistar control EVs at 28 weeks as determined by qRT-PCR (using an unpaired t-test). Heatmap summarizing this data is shown in Figure 3C. All data in the figure depict mean +/- SEM.

Figure S5: Time-course of miRNA dysregulation in circulating EVs during pathogenesis in *db/db* mice. Relative abundance of several miRNAs that were found to be dysregulated in *db/db* EVs at 14 weeks (see Figure 3A, B) assessed by qRT-PCR in independent samples. Expression was assessed at 4-6, 8 and 14 weeks in EVs isolated from control and *db/db* mice. Several up-

regulated miRNAs were found to be elevated as early as 8 weeks (top), while down-regulated miRNAs were primarily down-regulated at late stages (i.e. 14 weeks) (bottom left). Several miRNAs were not validated in these samples (bottom right). n=4-5 for each time-point and genotype. * and ** indicate p<0.05 and p<0.01, respectively, for *db/db* vs. control *db/+* at the specified timepoint using an unpaired t-test. All data in the figure depict mean +/- SEM.

Figure S6: MiR-25 and miR-92a are not differentially expressed across tissues in *db/db* mice. Expression of miR-25-3p and miR-92a-3p by qRT-PCR analysis of several tissues in *db/db* mice and controls at 14 weeks of age. Data is relative to the mean of the control group for each tissue sample. No significant differences in expression were identified using unpaired t-tests for each tissue. All data in the figure depict mean +/- SEM.

Figure S7: MiR-30d and miR-30e are predicted to regulated fatty acid biosynthesis and metabolism genes in mouse. Pathway analysis (DIANA-miRPath v3.0 with Tarbase v7.0) of mouse miR-30d and miR-30e target genes reveals a significant enrichment in fatty acid biosynthesis and fatty acid metabolism genes.

Figure S8: MiR-30 regulates predicted target genes involved in fatty acid biosynthesis and metabolism. (A) Expression of predicted miR-30 target genes in HUVEC transfected with control or miR-30e mimic under control and palmitate stimulated conditions. *, ** and *** indicate p<0.05, p<0.01 and p<0.001, respectively, for the specified comparison using ANOVA and the two-stage linear step-up procedure of Benjamini, Krieger and Yekutieli for multiple comparison testing. **(B)** Expression of predicted miR-30 target genes in ECs transfected with control or miR-30 family inhibitors under control and palmitate stimulated conditions. *, ** and *** indicate p<0.05, p<0.01 and p<0.001, respectively, for the specified comparison using ANOVA and the two-stage linear step-up procedure of Benjamini, Krieger and Yekutieli for multiple comparison testing. All data in the figure depict mean +/- SEM.

Figure S9: MiR-30e enhances fatty acid storage in ECs and does not affect ER stress. (A) Representative images (left) and quantification (right) of lipid droplet formation (BODIPY staining) in cells transfected with control or miR-30e mimic under basal, palmitate- and oleate-stimulated conditions. BODIPY staining was quantified in 4 individual cells in 3 fields of view across 2 independent experiments. Scale bar = 30 μm. *, ** and *** indicates p<0.05, p<0.01 and p<0.001, respectively, for the specified comparison using ANOVA with Holm-Sidak multiple comparisons test. **(B)** Measurement of the ER stress marker, spliced *XBP1*, by qRT-PCR in cells transfected with control or miR-30e mimic (left) or control or miR-30 family inhibitor (right) in the presence or absence of palmitate stimulation. No significant differences were observed using ANOVA with Holm-Sidak multiple comparison test. ns = not significant. All data in the figure depict mean +/- SEM.

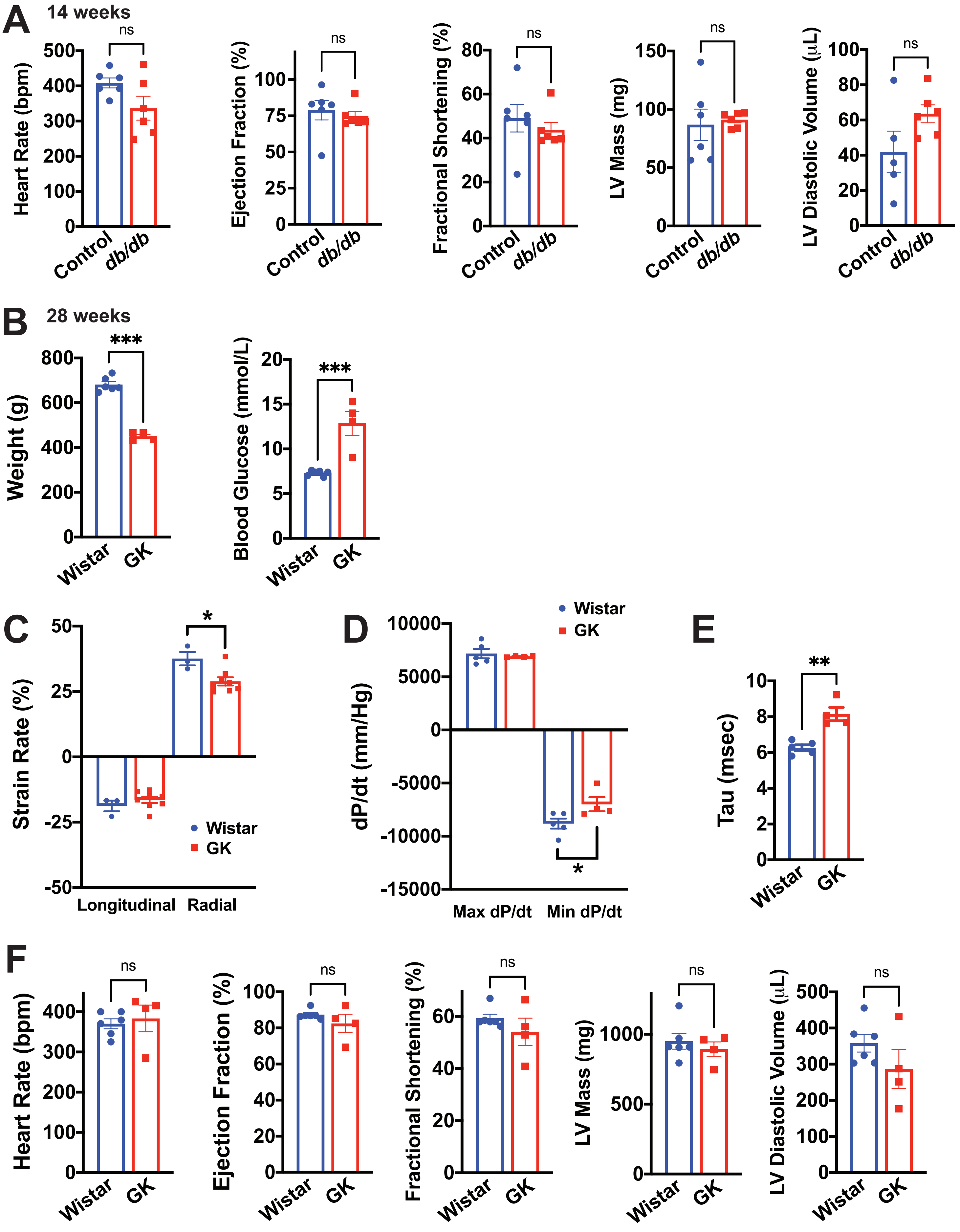
A
14 weeks
Heart Rate (bpm)
Ejection Fraction (%)
Fractional Shortening (%)
LV Mass (mg)
LV Diastolic Volume (μL)
ns
Control
db/db
B
28 weeks
Weight (g)
Blood Glucose (mmol/L)
***
Wistar
GK
C
Strain Rate (%)
*
Longitudinal
Radial
D
dP/dt (mm/Hg)
Max dP/dt
Min dP/dt
E
Tau (msec)
**
F
Heart Rate (bpm)
Ejection Fraction (%)
Fractional Shortening (%)
LV Mass (mg)
LV Diastolic Volume (μL)

Figure S1

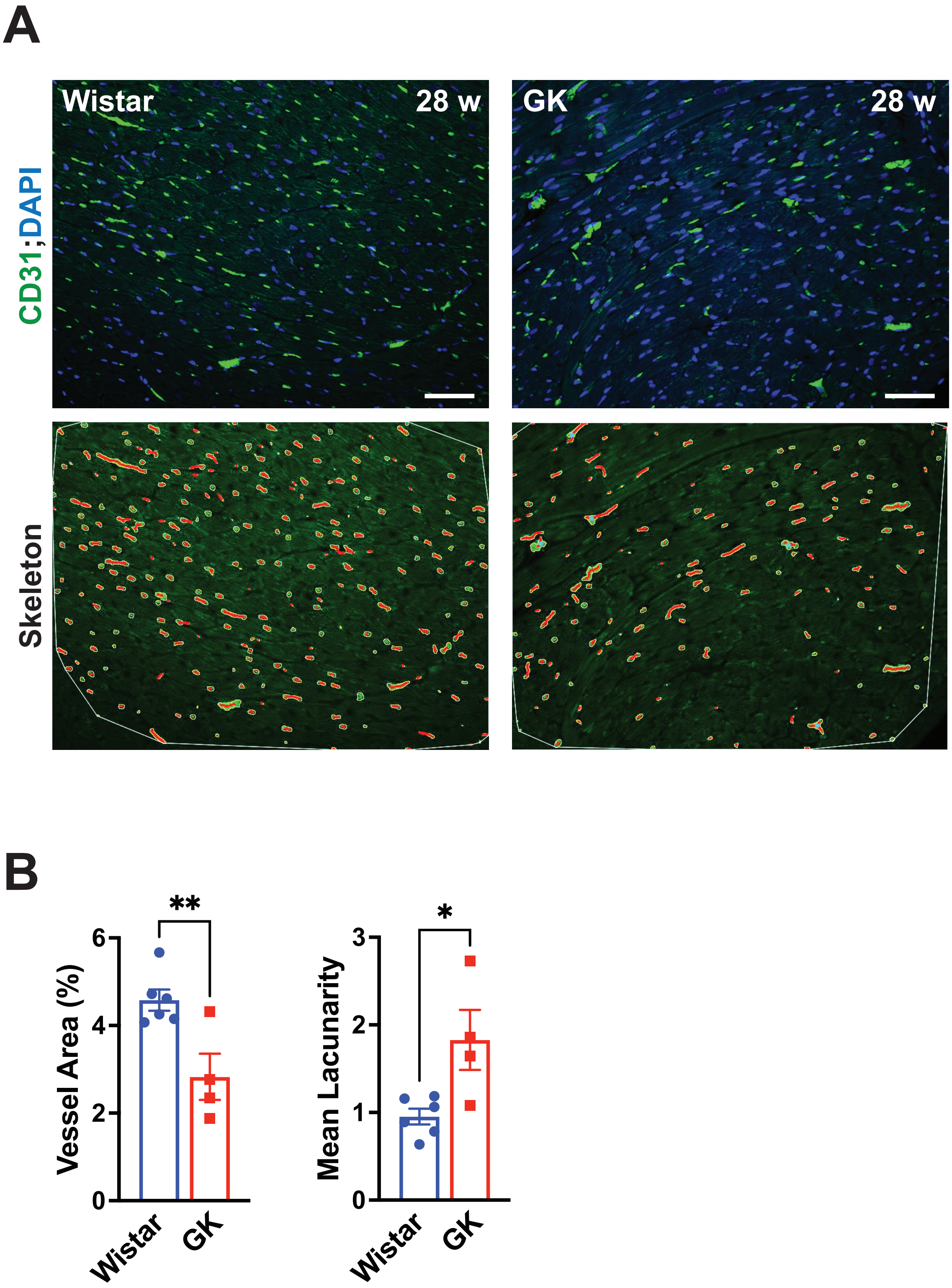

A
Wistar
28 w
GK
28 w
CD31;DAPI
Skeleton
B
**
*
6
4
2
0
Vessel Area (%)
Wistar
GK
3
2
1
0
Mean Lacunarity
Wistar
GK

Figure S2

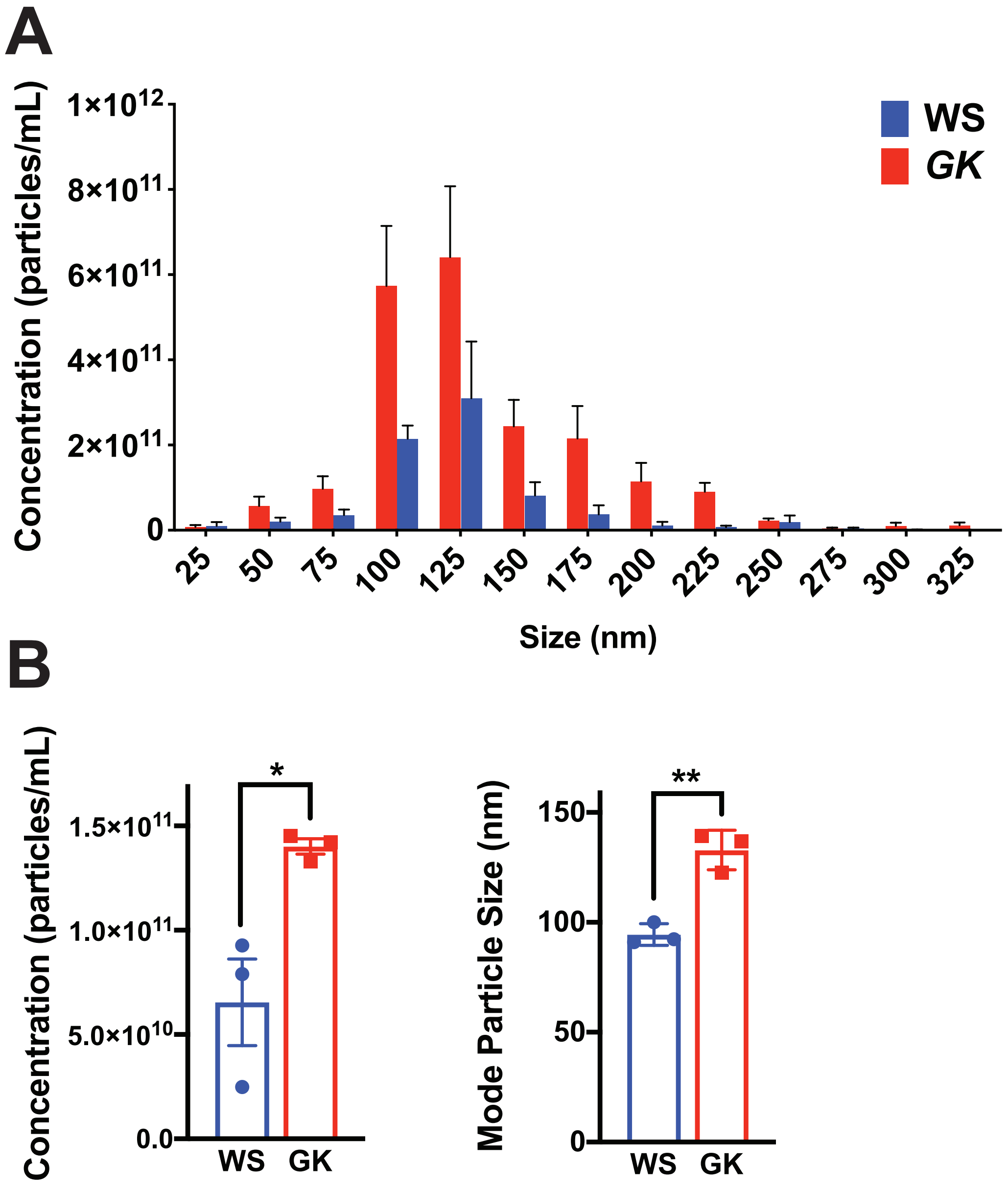
A
Concentration (particles/mL)
1×10^12
8×10^11
6×10^11
4×10^11
2×10^11
0
WS
GK
25
50
75
100
125
150
175
200
225
250
275
300
325
Size (nm)
B
Concentration (particles/mL)
1.5×10^11
1.0×10^11
5.0×10^10
0.0
*
WS GK
Mode Particle Size (nm)
150
100
50
0
**
WS GK

Figure S3

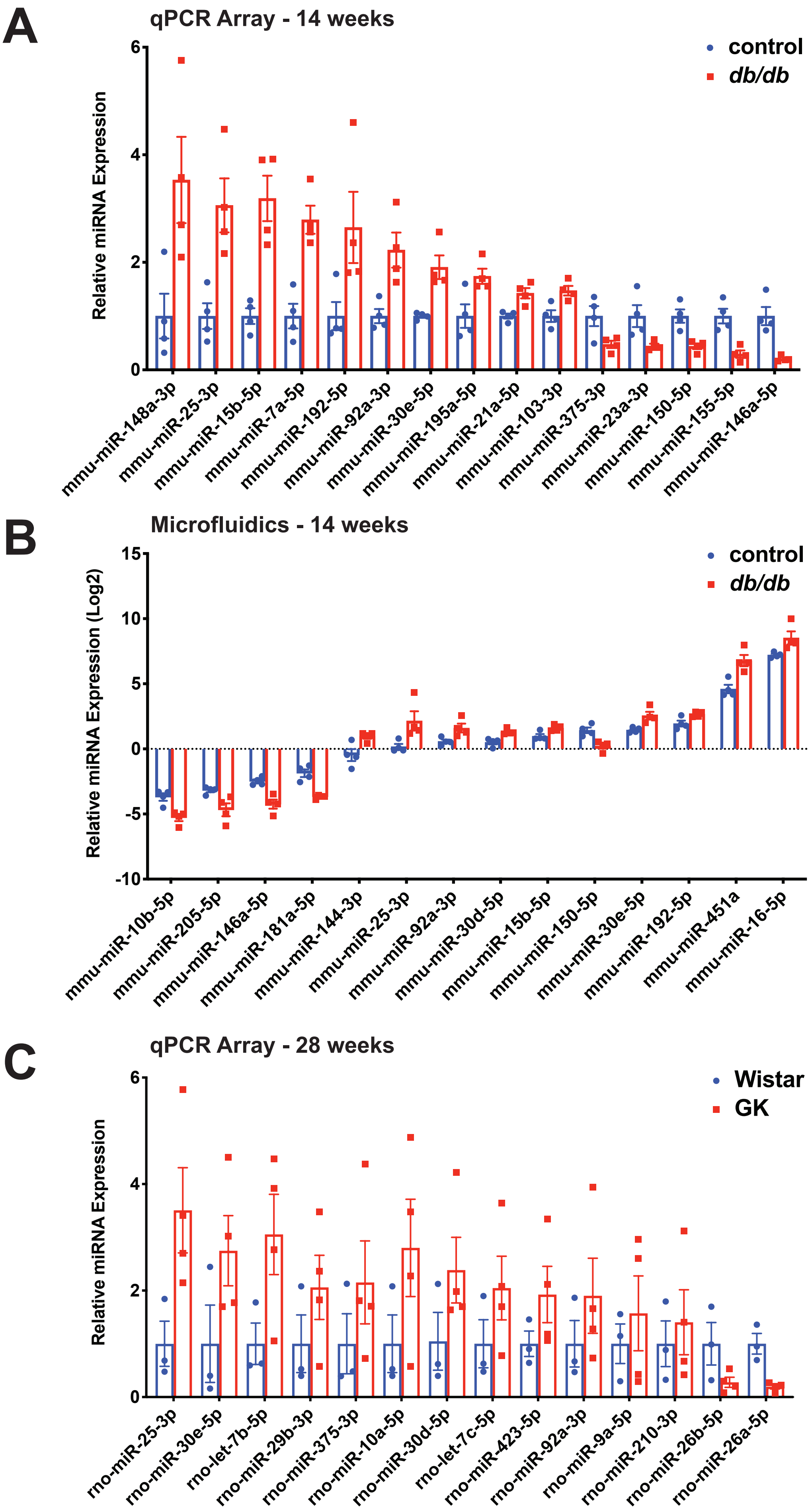

A
qPCR Array - 14 weeks
control
db/db
Relative miRNA Expression
0
2
4
6
mmu-miR-148a-3p
mmu-miR-25-3p
mmu-miR-15b-5p
mmu-miR-7a-5p
mmu-miR-192-5p
mmu-miR-92a-3p
mmu-miR-30e-5p
mmu-miR-195a-5p
mmu-miR-21a-5p
mmu-miR-103-3p
mmu-miR-375-3p
mmu-miR-23a-3p
mmu-miR-150-5p
mmu-miR-155-5p
mmu-miR-146a-5p
B
Microfluidics - 14 weeks
control
db/db
Relative miRNA Expression (Log2)
15
10
5
0
-5
-10
mmu-miR-10b-5p
mmu-miR-205-5p
mmu-miR-146a-5p
mmu-miR-181a-5p
mmu-miR-144-3p
mmu-miR-25-3p
mmu-miR-92a-3p
mmu-miR-30d-5p
mmu-miR-15b-5p
mmu-miR-150-5p
mmu-miR-30e-5p
mmu-miR-192-5p
mmu-miR-451a
mmu-miR-16-5p
C
qPCR Array - 28 weeks
Wistar
GK
Relative miRNA Expression
0
2
4
6
rno-miR-25-3p
rno-miR-30e-5p
rno-let-7b-5p
rno-miR-29b-3p
rno-miR-375-3p
rno-miR-10a-5p
rno-miR-30d-5p
rno-let-7c-5p
rno-miR-423-5p
rno-miR-92a-3p
rno-miR-9a-5p
rno-miR-210-3p
rno-miR-26b-5p
rno-miR-26a-5p

Figure S4

miRNAs induced in EVs during HFpEF pathogenesis:

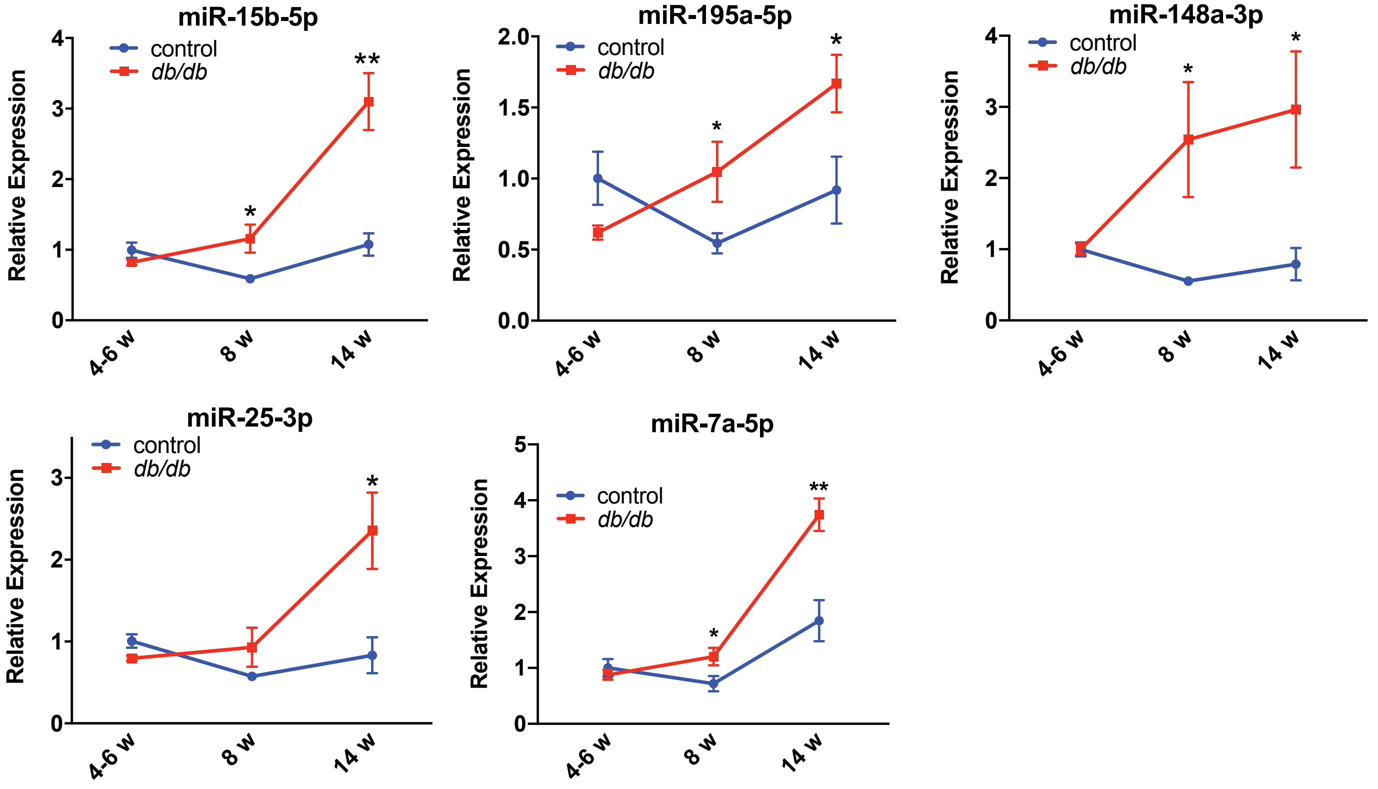

miRNAs down-regulated in EVs during HFpEF pathogenesis:

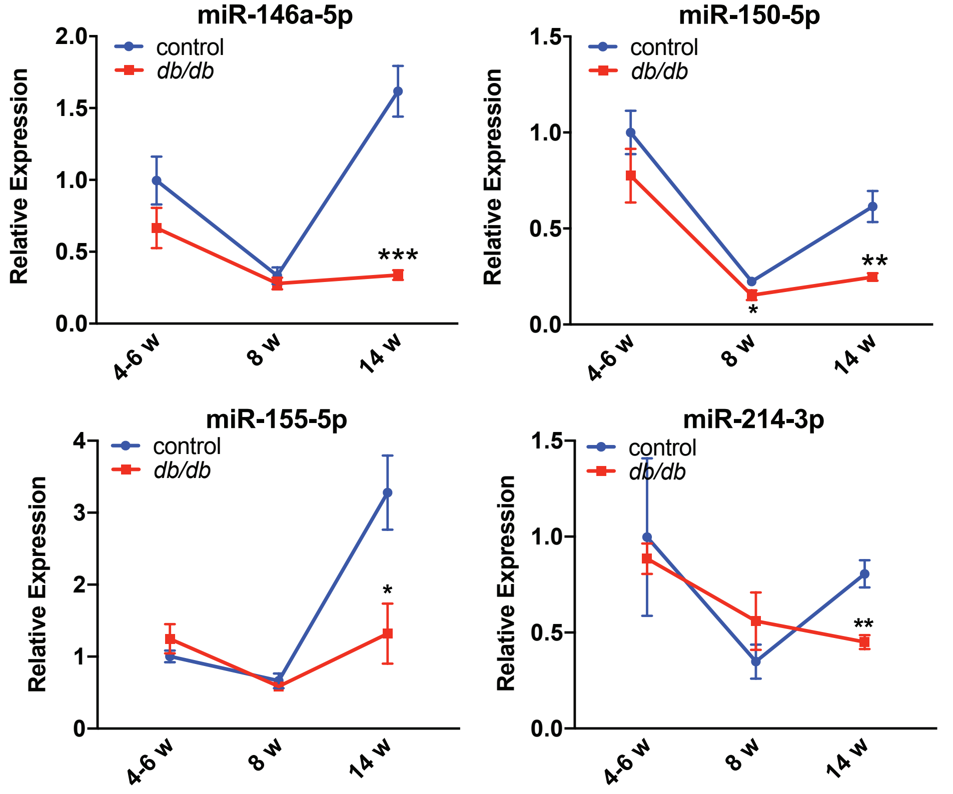

miRNAs that failed to validate:

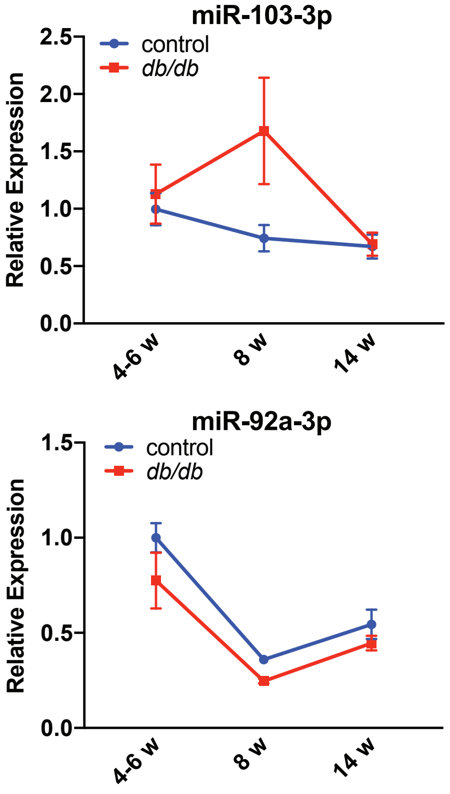

Figure S5

14 weeks

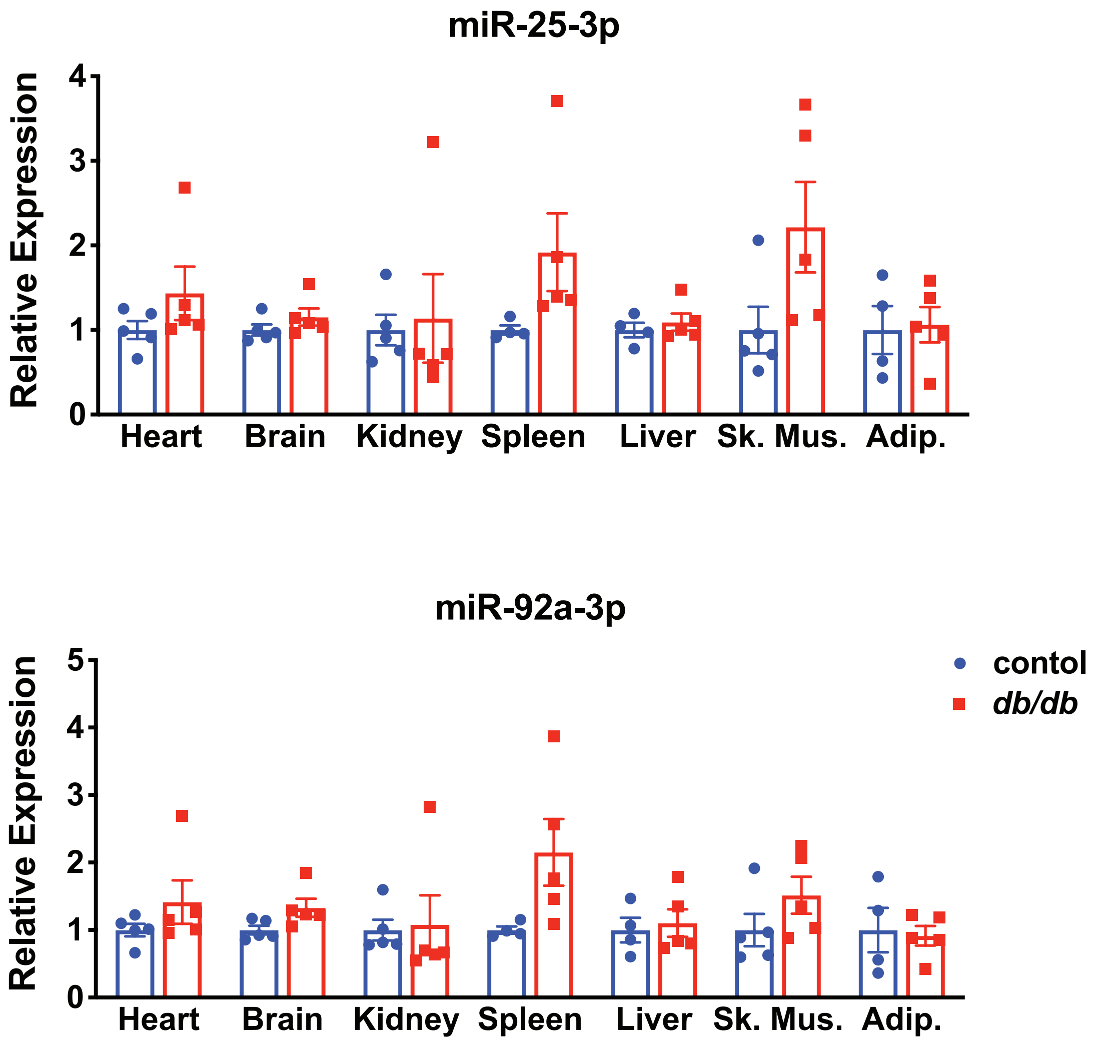

Figure S6

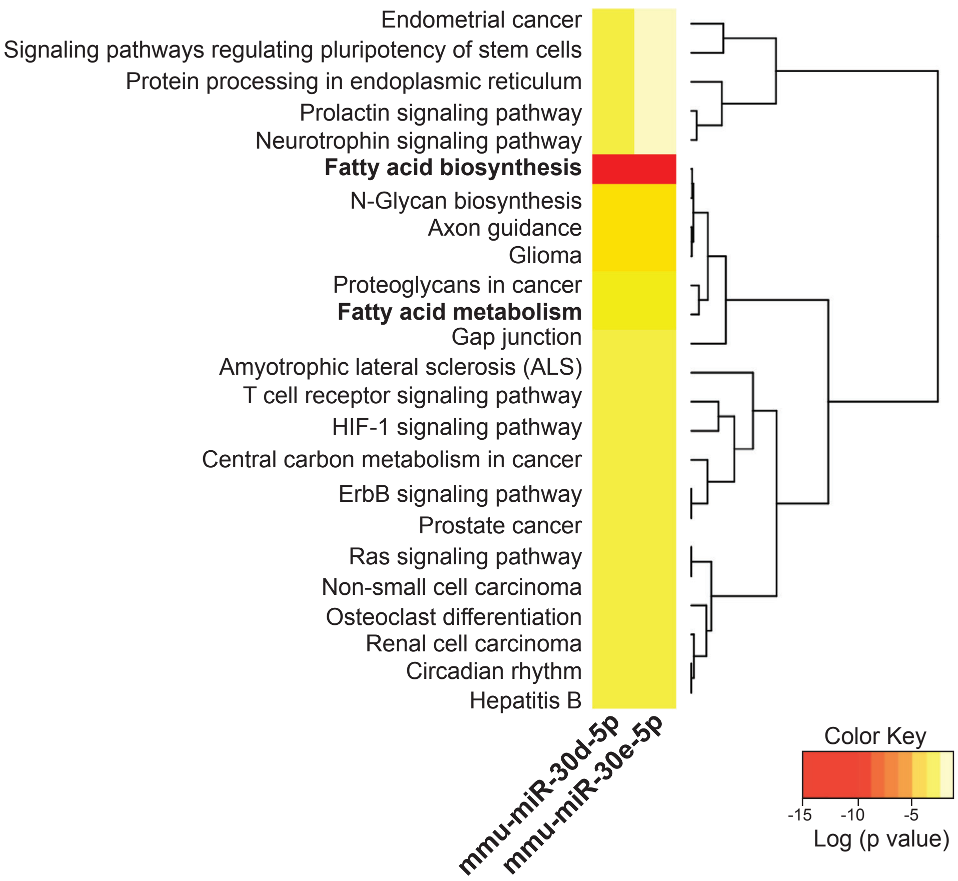
Endometrial cancer
Signaling pathways regulating pluripotency of stem cells
Protein processing in endoplasmic reticulum
Prolactin signaling pathway
Neurotrophin signaling pathway
Fatty acid biosynthesis
N-Glycan biosynthesis
Axon guidance
Glioma
Proteoglycans in cancer
Fatty acid metabolism
Gap junction
Amyotrophic lateral sclerosis (ALS)
T cell receptor signaling pathway
HIF-1 signaling pathway
Central carbon metabolism in cancer
ErbB signaling pathway
Prostate cancer
Ras signaling pathway
Non-small cell carcinoma
Osteoclast differentiation
Renal cell carcinoma
Circadian rhythm
Hepatitis B
mmu-miR-30d-5p
mmu-miR-30e-5p
Color Key
-15
-10
-5
Log (p value)

Figure S7

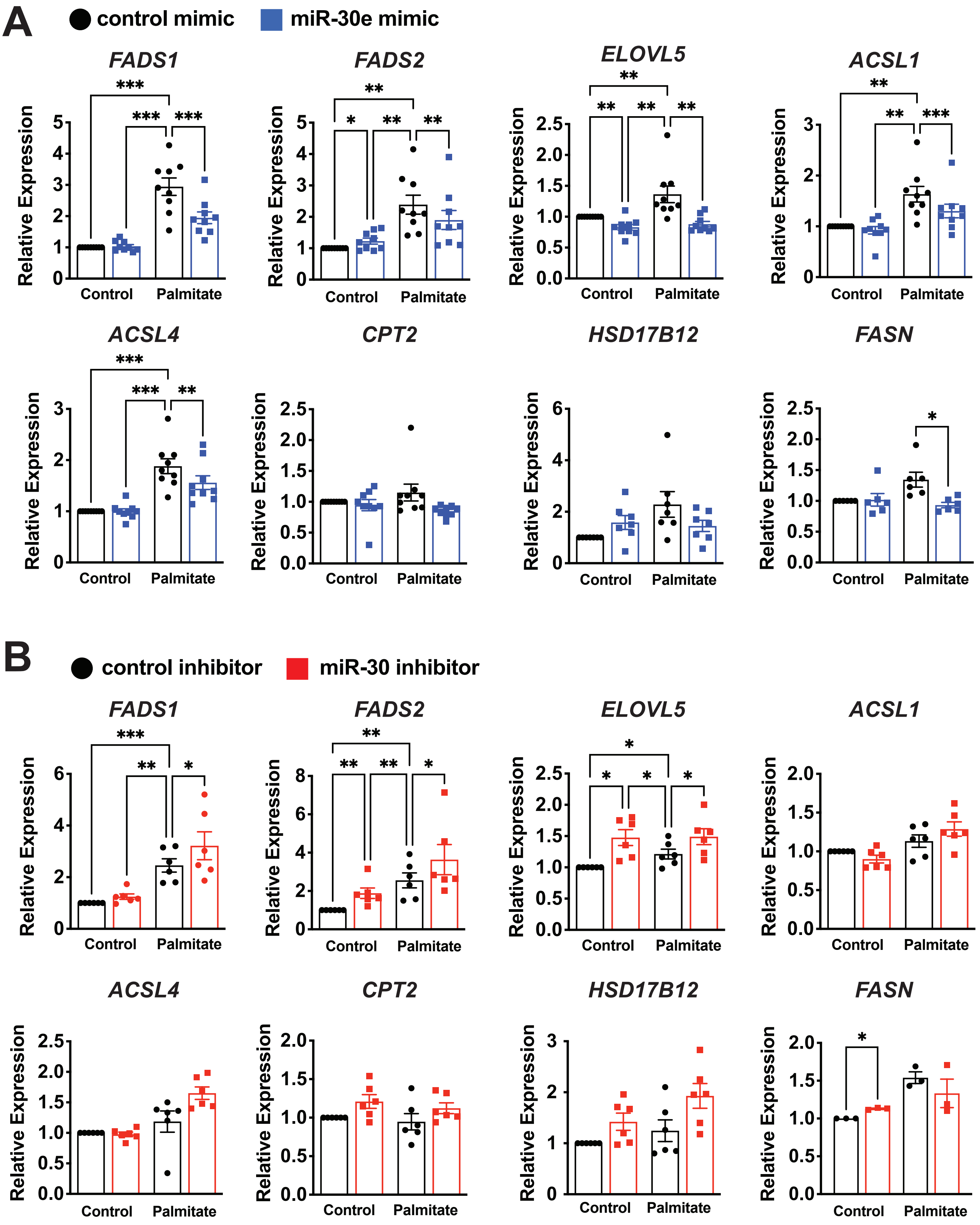
A
control mimic
miR-30e mimic
FADS1
FADS2
ELOVL5
ACSL1
ACSL4
CPT2
HSD17B12
FASN
Relative Expression
Control
Palmitate
B
control inhibitor
miR-30 inhibitor
FADS1
FADS2
ELOVL5
ACSL1
ACSL4
CPT2
HSD17B12
FASN
Relative Expression
Control
Palmitate

Figure S8

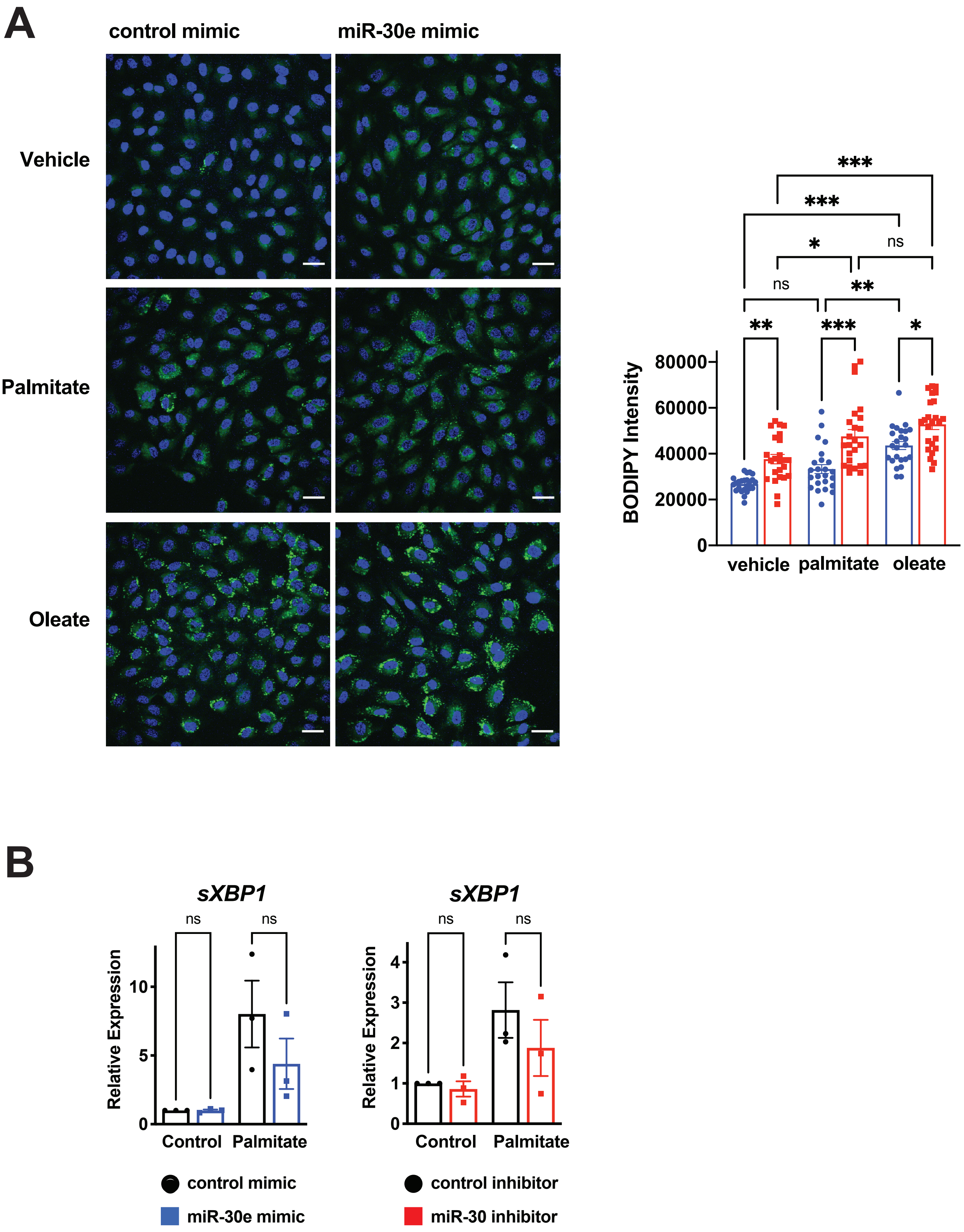
A
control mimic
miR-30e mimic
Vehicle
Palmitate
Oleate
BODIPY Intensity
80000
60000
40000
20000
0
vehicle
palmitate
oleate
***
***
*
ns
ns
**
**
***
*
B
sXBP1
Relative Expression
10
5
0
Control
Palmitate
ns
ns
control mimic
miR-30e mimic
sXBP1
Relative Expression
4
3
2
1
0
Control
Palmitate
ns
ns
control inhibitor
miR-30 inhibitor

Figure S9
